# Treatment with soluble CD24 attenuates COVID-19-associated systemic immunopathology

**DOI:** 10.1186/s13045-021-01222-y

**Published:** 2022-01-10

**Authors:** No-Joon Song, Carter Allen, Anna E. Vilgelm, Brian P. Riesenberg, Kevin P. Weller, Kelsi Reynolds, Karthik B. Chakravarthy, Amrendra Kumar, Aastha Khatiwada, Zequn Sun, Anjun Ma, Yuzhou Chang, Mohamed Yusuf, Anqi Li, Cong Zeng, John P. Evans, Donna Bucci, Manuja Gunasena, Menglin Xu, Namal P. M. Liyanage, Chelsea Bolyard, Maria Velegraki, Shan-Lu Liu, Qin Ma, Martin Devenport, Yang Liu, Pan Zheng, Carlos D. Malvestutto, Dongjun Chung, Zihai Li

**Affiliations:** 1 The Pelotonia Institute for Immuno-Oncology, The Ohio State University James Comprehensive Cancer Center, 460 W. 12th Ave, Columbus, OH 43210 USA; 2 Department of Biomedical Informatics, The Ohio State University College of Medicine, Columbus, OH USA; 3 Department of Pathology, The Ohio State University College of Medicine, Columbus, OH USA; 4 The Ohio State University College of Medicine, Columbus, OH USA; 5 The Ohio State University Comprehensive Cancer Center, Columbus, OH USA; 6 Department of Public Health Sciences, Medical University of South Carolina, Charleston, SC USA; 7 Center for Retrovirus Research and Department of Veterinary Biosciences, The Ohio State University, Columbus, OH USA; 8 Department of Microbial Infection and Immunity, The Ohio State University College of Medicine, Columbus, OH USA; 9 Department of Veterinary Biosciences, The Ohio State University College of Veterinary Medicine, Columbus, OH USA; 10 Department of Internal Medicine, The Ohio State University College of Medicine, Columbus, OH USA; 11 OncoC4, Rockville, MD USA

**Keywords:** COVID-19, CD24Fc, Soluble CD24, Immunophenotyping, Cytokine score

## Abstract

**Background:**

Severe Acute Respiratory Syndrome Coronavirus 2 (SARS-CoV-2) causes coronavirus disease 2019 (COVID-19) through direct lysis of infected lung epithelial cells, which releases damage-associated molecular patterns and induces a pro-inflammatory cytokine milieu causing systemic inflammation. Anti-viral and anti-inflammatory agents have shown limited therapeutic efficacy. Soluble CD24 (CD24Fc) blunts the broad inflammatory response induced by damage-associated molecular patterns via binding to extracellular high mobility group box 1 and heat shock proteins, as well as regulating the downstream Siglec10-Src homology 2 domain–containing phosphatase 1 pathway. A recent randomized phase III trial evaluating CD24Fc for patients with severe COVID-19 (SAC-COVID; NCT04317040) demonstrated encouraging clinical efficacy.

**Methods:**

Using a systems analytical approach, we studied peripheral blood samples obtained from patients enrolled at a single institution in the SAC-COVID trial to discern the impact of CD24Fc treatment on immune homeostasis. We performed high dimensional spectral flow cytometry and measured the levels of a broad array of cytokines and chemokines to discern the impact of CD24Fc treatment on immune homeostasis in patients with COVID-19.

**Results:**

Twenty-two patients were enrolled, and the clinical characteristics from the CD24Fc vs. placebo groups were matched. Using high-content spectral flow cytometry and network-level analysis, we found that patients with severe COVID-19 had systemic hyper-activation of multiple cellular compartments, including CD8^+^ T cells, CD4^+^ T cells, and CD56^+^ natural killer cells. Treatment with CD24Fc blunted this systemic inflammation, inducing a return to homeostasis in NK and T cells without compromising the anti-Spike protein antibody response. CD24Fc significantly attenuated the systemic cytokine response and diminished the cytokine coexpression and network connectivity linked with COVID-19 severity and pathogenesis.

**Conclusions:**

Our data demonstrate that CD24Fc rapidly down-modulates systemic inflammation and restores immune homeostasis in SARS-CoV-2-infected individuals, supporting further development of CD24Fc as a novel therapeutic against severe COVID-19.

**Supplementary Information:**

The online version contains supplementary material available at 10.1186/s13045-021-01222-y.

## Background

The pathogenesis associated with Severe Acute Respiratory Syndrome Coronavirus 2 (SARS-CoV-2) is a multistep process starting with the infection of angiotensin-converting enzyme 2 (ACE2)-expressing lung epithelial cells [1]. Following infection, unconstrained viral replication leads to cell lysis and the release of damage-associated molecular patterns (DAMPs) [2, 3]. Recognition of these molecules by neighboring cells produces a pro-inflammatory milieu by releasing cytokines (such as interleukin IL-6 and IL-10), which recruit and activate monocytes, macrophages, and T cells [4]. In severe COVID-19, this pro-inflammatory feedback loop results in a persistent and harmful response that leads to structural damage of the lung. The resulting cytokine storm can lead to acute respiratory distress syndrome (ARDS), multi-organ failure, and death [5].

Even though COVID-19 messenger RNA (mRNA) vaccines have shown great success in preventing severe disease [6], recent reports suggest that SARS-CoV-2 variants may escape or subvert the immune response induced by existing vaccines [7]. Breakthrough infections following full vaccination can occur [8], especially in immunocompromised individuals [9], requiring urgent development of effective therapeutic agents against this disease. Interim results from the Solidarity trial (NCT04315948) indicate that several repurposed interventions do not significantly alter COVID-19 morbidity and mortality [10]. Other approaches, including cytokines and convalescent plasma, have also been largely ineffective [11, 12]. The anti-inflammatory glucocorticoid dexamethasone and the protease inhibitor ritonavir represent the few interventions shown to reduce mortality in patients with critical-to-severe COVID-19 ([13] and NCT04960202), especially in combination with monoclonal antibodies against the SARS-CoV2 spike protein [14].

We previously demonstrated that CD24-deficient mice display increased inflammation and death in response to damage triggered by radiation and other means [15]. CD24 is an important checkpoint molecule for controlling the innate immune response [16]; it binds to extracellular high-mobility group box 1 (HMGB1) and heat shock proteins, as well as the downstream Siglec10-SHP1 pathway to blunt NF-κB activation [15]. Soluble CD24 (CD24Fc), which is linked to the Fc domain of human IgG1, was developed to treat inflammatory conditions in patients. CD24Fc treatment can attenuate inflammation associated with viral infections, autoimmunity, and graft-versus-host diseases [17–19]. We launched and completed a phase III clinical trial to determine whether CD24Fc provides therapeutic benefit to patients with severe COVID-19. Interim analysis of results from 197 patients (placebo treatment, *n* = 98; CD24Fc treatment, *n* = 99) found a statistically significant improvement in clinical status in patients treated with CD24Fc versus placebo (*p* = 0.005; HR = 1.61, 95% CI: 1.16 to 2.23) over the 28-day study period with median times to clinical recovery of 6 days for CD24Fc compared to 10 days for placebo (These results are under review at *Lancet Infectious Diseases*: “Therapeutic Efficacy and Safety of CD24Fc in Hospitalized Patients with COVID-19,” by Welker et al*.*). In the current study, we performed extensive correlative analysis in 24 patients enrolled at a single academic setting (i.e., The Ohio State University Wexner Medical Center). We compared blood samples from COVID-19 patients before (“baseline”) and after treatment with CD24Fc or placebo and compared to healthy donor (HD) controls. We examined dynamic changes in peripheral blood mononuclear cells (PBMCs) and systemic cytokine and chemokine levels. We demonstrated that CD24Fc reversed the inflammatory hallmarks associated with severe COVID-19, including cytokine storm and immune hyperactivation.

## Methods

### Patients and trial procedure

This study included samples from all patients enrolled in NCT04317040 at The Ohio State University Wexner Medical Center. Patients eligible for this trial were hospitalized with COVID-19, requiring supplemental oxygen but not mechanical ventilation and had a prior positive SARS-CoV-2 PCR test. Enrolled patients were randomized in a double-blinded fashion by the hospital pharmacist to receive either a single dose of CD24Fc antibody (480 mg IV infusion) or placebo control (IV saline). Peripheral blood samples were collected from patients prior to drug infusion (D1) and at subsequent time points 1, 3, 7, 14, and 28 days after drug infusion (D2, D4, D8, D15, and D29). Patients were monitored until D29, after which they completed the study endpoint. Samples from D1, D2, D4, and D8 were evaluated as only 2 samples were acquired for D15, and we did not obtain D29 sample. Pertinent patient clinical information was abstracted from the internal electronic medical record database, including demographic data, medical history, clinical laboratory findings, and treatment regimen for COVID-19 during hospital stay (Additional file 1: Table S1). All enrolled patients were able to complete the study endpoint with no death during study enrollment in either group. After enrollment and completion of the study period, two patients were excluded from the analysis. One exclusion was due to a diagnosis of chronic lymphocytic leukemia (CLL), which confounded the subsequent immunological analyses. Another exclusion occurred with a patient who received an infusion but was discharged before any post-infusion peripheral blood sample could be collected; hence, no comparative analysis could be made using this patient. Patient characteristics were clinically matched between the two groups. All patients enrolled in the study received a treatment regimen for COVID-19 by hospital care teams regardless of their placebo/CD24Fc treatment status. Patients were randomized in a double-blind fashion into CD24Fc antibody treatment group (*n* = 10) or placebo control group (*n* = 12).

### PBMC collection and flow cytometry staining

Samples for this study were collected from patients enrolled in clinical trial NCT04317040. We analyzed samples from 22 patients hospitalized at The Ohio State University Wexner Medical Center with severe COVID-19. Peripheral blood mononuclear cells (PBMCs) were isolated per the manufacturer’s protocol using CPT tubes (BD Bioscience). Healthy donor (HD) PMBCs were obtained from STEMCELL Technologies™. We utilized a 36-color flow cytometry panel (Additional file 1: Table S2, developed by Cytek [20]) to distinguish immune populations; we developed a 25-color-panel to study the activation status of CD8^+^, CD4^+^, and CD56^+^ subsets. For the 25-color-panel, surface markers were stained in 4 °C for 1 h, and FOXP3/Transcription Factor Staining Buffer Set (eBioscience™) was used per manufacturers recommendation to perform intracellular staining. Cells were analyzed using the Cytek Aurora system.

### Virus neutralization assay

Virus was produced as previously described [21] and incubated with COVID-19 patient sera for 1 h at 37 °C. Virus was then overlaid onto ACE2-expressing 293 T cells for 6 h. Gaussia luciferase (Gluc) activity was measured 24, 48, and 72 h after infection.

### Cytokine and chemokine assay

Plasma samples were processed using multiplexed ELISA-based platform Quantibody® Human Inflammation Array 3 (RayBiotech QAH-INF-3) in accordance with the manufacturer’s protocol. Slides were shipped to the manufacturer site for scanning and data extraction services. Raw optical data were analyzed using the manufacturer’s analysis tool to construct standard curves and determine absolute cytokine concentrations (Additional file 2: Table S3). Cytokines for which standards did not yield good standard curve fit or that were undetectable were excluded (IFNγ, IL1rα, IL2, IL13, MCP-1, TNFα, TNFβ, IL-11, IL-12p70, IL-17A). Seven of these cytokines were detected using an alternative method. Specifically, cytokines IFNγ, IL1rα, IL2, IL13, MCP-1, TNFα, and IL-12p70 were measured by Luminex analysis. For that, plasma samples were sent to EVE Technologies that performed the assay and provided cytokine concentration data (Additional file 3: Table S4).

### Flow cytometry data analysis

We integrated flow cytometry marker data from all samples, and arcsinh scaling was applied using OMIQ (https://www.omiq.ai/). Then, we visualized cells in a reduced two-dimensional space using the Uniform Manifold Approximation and Projection (UMAP) algorithm implemented in the R package uwot [22]. We adopted a multivariate t-mixture model to cluster cells based on the normalized multivariate flow cytometry marker expression [23]. For each data set, we chose the optimal number of cell clusters by selecting the model with the minimum Bayesian information criterion (BIC) score [24]. Then, we annotated cell types by visually investigating heatmaps of median marker expressions across clusters and expressions of these markers on the UMAP space.

### Immune cell activation score construction

To measure activation, we defined a cell-level immune cell activation score for each flow cytometry data set. We selected a subset of immune cell activation markers from the panel [25, 26] and ran a principal component analysis (PCA) comparing cells from HD and baseline (Day 1) COVID-19 patients using these activation markers as features. We used the first principal component (PC1) as an activation score to reflect the differences in immune cell activation between groups. The loadings of each pre-selected activation marker onto PC1 were used as coefficients to compute an activation score for COVID-19 patients after baseline.

### Cytokine score construction

To construct the cytokine score, we implemented a weighted sum approach, motivated by the polygenic risk score calculation in the genome-wide association study (GWAS). First, we fit a generalized linear mixed model (GLMM) of each cytokine measurement (base 10 log-transformed) on treatment, time, treatment*time, age, sex, and race as fixed-effect terms, along with subject-level random effect terms. To compare longitudinal patterns across groups, each cytokine had its group-specific baseline mean adjusted to match the overall mean at D1, and consequent time points are normalized accordingly, followed by scaling-by-row. Second, the *p*-value for evaluating the overall difference in trends between CD24Fc and placebo groups across all the time points was calculated using the Kenward-Roger method [27]. Finally, we obtained the weighted sum of cytokine measurements using the -2 log-transformed *p*-value for the trend difference as weights, motivated by Fisher’s method. We validated the above approach using the PCA and autoencoder approaches [28].

### Network-level analysis of cytokine data

We first calculated Pearson correlation coefficients between cytokines (base 10 log-transformed). Then, we constructed a network, where a node represents a cytokine, and an edge between two nodes was built if the corresponding absolute correlation coefficient is larger than 0.4, a cutoff that is usually considered to be a moderate correlation [29]. The weight of an edge represents the corresponding correlation coefficient. A network was built via the MetScape [30] (version 3.1.3) application in Cytoscape [31] (version 3.8.0). We evaluated the network structure and the importance of each node in the network based on an eigenvector centrality (EC) score [32] using the CytoNCA [33] (version 2.1.6) application in Cytoscape (version 3.8.0). Nodes with larger EC scores can be considered of higher importance.

### Treatment group determination

The treatment group (control vs. CD24Fc) was determined by the post-infusion sera to absorb anti-CD24 antibody for staining of human CD24^+^ cells by flow cytometry. The patient group on the CD24Fc arm was further confirmed using CD24Fc ELISA (capture antibody: purified anti-human CD24, Clone ML5, BD bioscience, Cat#555,426. San Jose, CA).

### Bioinformatics and statistical analysis

Bioinformatic analyses were performed as previously described [23, 25, 26, 28–33]. Flow cytometry data were preprocessed using the OMIQ software, visualized using the UMAP algorithm, and analyzed using a multivariate t-mixture model [23]. The immune cell activation score was constructed by aggregating pre-selected activation markers [25, 26] using a PCA applied to the flow cytometry data of HD and baseline COVID-19 patients. Cytokine score was constructed using a weighted sum approach and validated using PCA and autoencoder approaches [28]. Network-level analysis of cytokine data was implemented by constructing a correlation network between cytokines and evaluating the network structure and importance of each node in the network based on an eigenvector centrality (EC) score [32]. Group comparisons were evaluated using independent sample t-test or Kruskal–Wallis test for continuous variables and Chi-squared test for categorical variables. Longitudinal analyses were implemented using GLMMs. In the longitudinal analyses, the overall differences in trends between CD24Fc and placebo groups across all the time points were evaluated using a GLMM of each measurement on treatment, time, treatment*time, age, sex, and race as fixed-effect terms, along with patient-level random intercepts. All data were analyzed using the R statistical package. All mixed models were fit using the lme4 package [34]. The *p*-value for evaluating the overall difference in trends between CD24Fc and placebo groups across all the time points was calculated using the Kenward-Roger method [27]. The observed values and trend lines are centered at the baseline.

## Results

### Impact on population dynamics of periperhal blood immune cells by CD24Fc

**Fig. 1:**
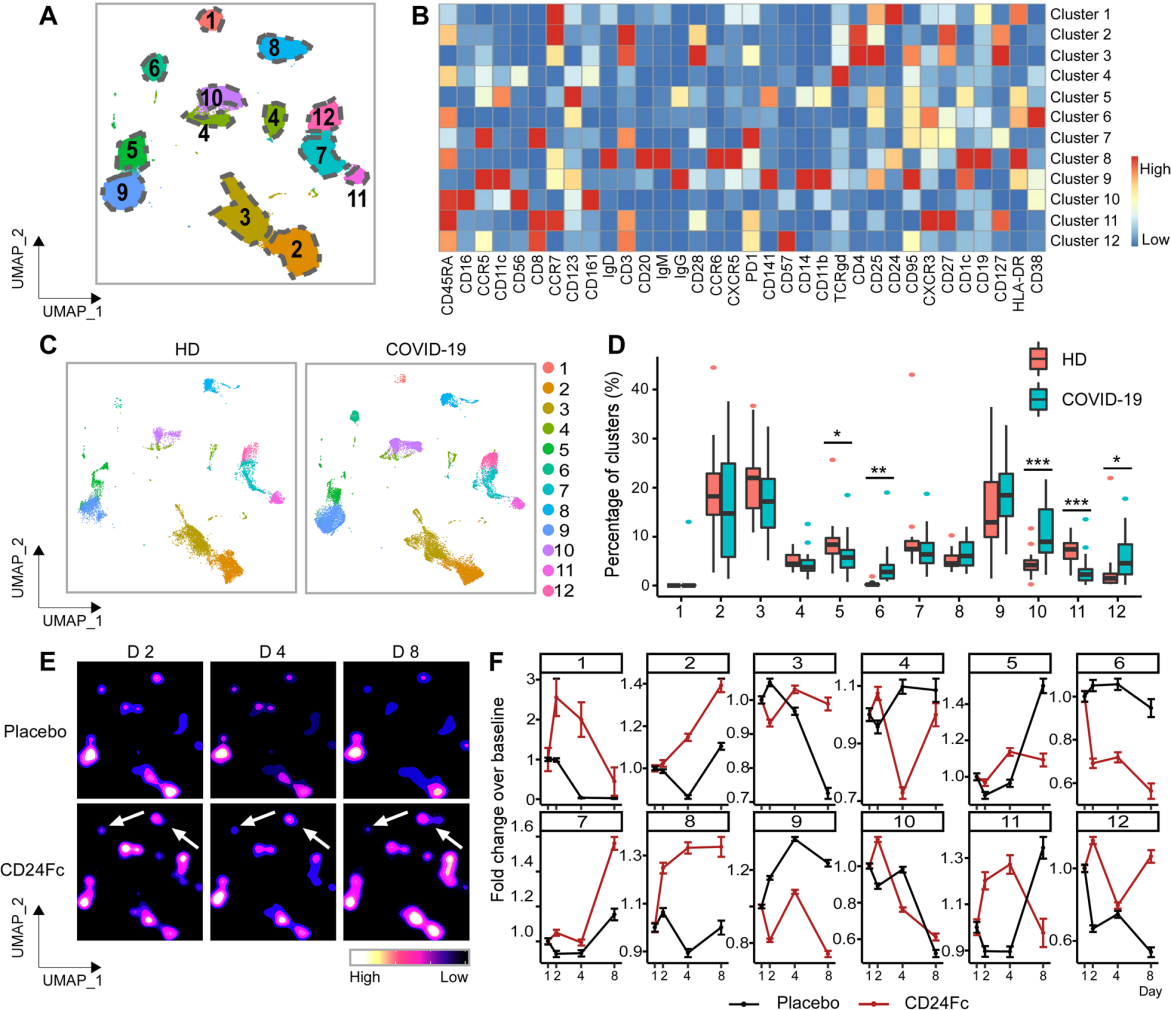
Population dynamics of peripheral blood mononuclear cells from healthy donors vs. patients with COVID-19 treated with placebo or CD24Fc. A total of 1,306,473 PBMCs from HD (*n* = 17) and COVID-19 patients (*n* = 22) were clustered using an unbiased multivariate *t*-mixture model, which identified 12 sub-clusters that reflect statistically distinct cell states. Visualization of the relative similarity of each cell and cell cluster on the two-dimensional UMAP space with a 10% downsampling (**A**). Cluster-by-marker heatmap characterizing the expression patterns of individual clusters (**B**). UMAP plots (**C**) and cluster frequencies (**D**) of HD vs. baseline COVID-19 patient samples (cluster 5, *p* = 0.03; cluster 6, *p* = 0.001; cluster 10, *p* < 0.001; cluster 11, *p* < 0.001; cluster 12, *p* = 0.01). Contour plots representing the density of cells throughout regions of the UMAP space from COVID-19 patients D2, D4, and D8 after CD24Fc vs. placebo treatment (**E**, white arrows indicate visual changes between CD24Fc vs. placebo contour plots). Cluster population dynamics as fold change over baseline for each group over time (**F**; *p* < 0.001 for cluster 1–12) (D2: placebo *n* = 12, CD24Fc *n* = 10; D4: placebo *n* = 11, CD24Fc *n* = 9; D8: placebo *n* = 4, CD24Fc *n* = 3). The *p*-value in **D** was calculated using the Wilcoxon rank-sum test. *p < 0.05; **p < 0.01; ***p < 0.001

We utilized a high dimensional spectral flow cytometry panel with an extensive array of immune population markers (Additional file 1: Table S2) to analyze the systemic effects of SARS-CoV-2 and CD24Fc treatment on PBMCs. Using an unbiased clustering approach based on a multivariate *t*-mixture model [23], we identified 12 statistically distinct clusters that we visualized in two dimensions using the UMAP algorithm (Fig. 1A). Using clustered heatmap analysis, we correlated expression intensity with clusters to annotate B cells (clusters 1, 6, 8), CD8^+^ T cells (clusters 7, 11, 12), CD4^+^ T cells (clusters 2, 3), γδ T cells (cluster 4), natural killer (NK) cells (cluster 10), and myeloid cells (clusters 5, 9) (Fig. 1B). Comparing systemic immune population dynamics (Fig. 1C, D), we found significant increases in plasma B cells (cluster 6; *p* < 0.01), NK cells (cluster 10; *p* < 0.001), and terminally differentiated CD8^+^ T cells (cluster 12; *p* < 0.05) in baseline (D1) COVID-19 patients vs. healthy donors (HD). Conversely, we found that HD samples were enriched for naïve CD8^+^ T cells (cluster 11; *p* < 0.001) and a subset of myeloid cells (cluster 5; *p* < 0.05). These initial findings are consistent with the established immunopathology of SARS-CoV-2 infection and the critical role of the adaptive immune system in viral pathogen response [35–38], thus validating our experimental approach.

We next used UMAP contour plots to investigate the effects of CD24Fc treatment on immune population dynamics over time (Fig. 1E, F). From baseline to D8, the CD24Fc group displayed a sharp and steady decline of plasma B cells (cluster 6), which coordinated with a proportional increase in mature B cells (cluster 8). The placebo group showed relatively stable cell proportions for these populations over the same time frame. There were no significant differences between the two groups in mounting an effective anti-Spike protein antibody response (Additional file 1: Fig. S1).

### CD24Fc treatment correlates with normalization of CD4^+^ and CD8^+^ T cells based on the changes of activation markers

**Fig. 2:**
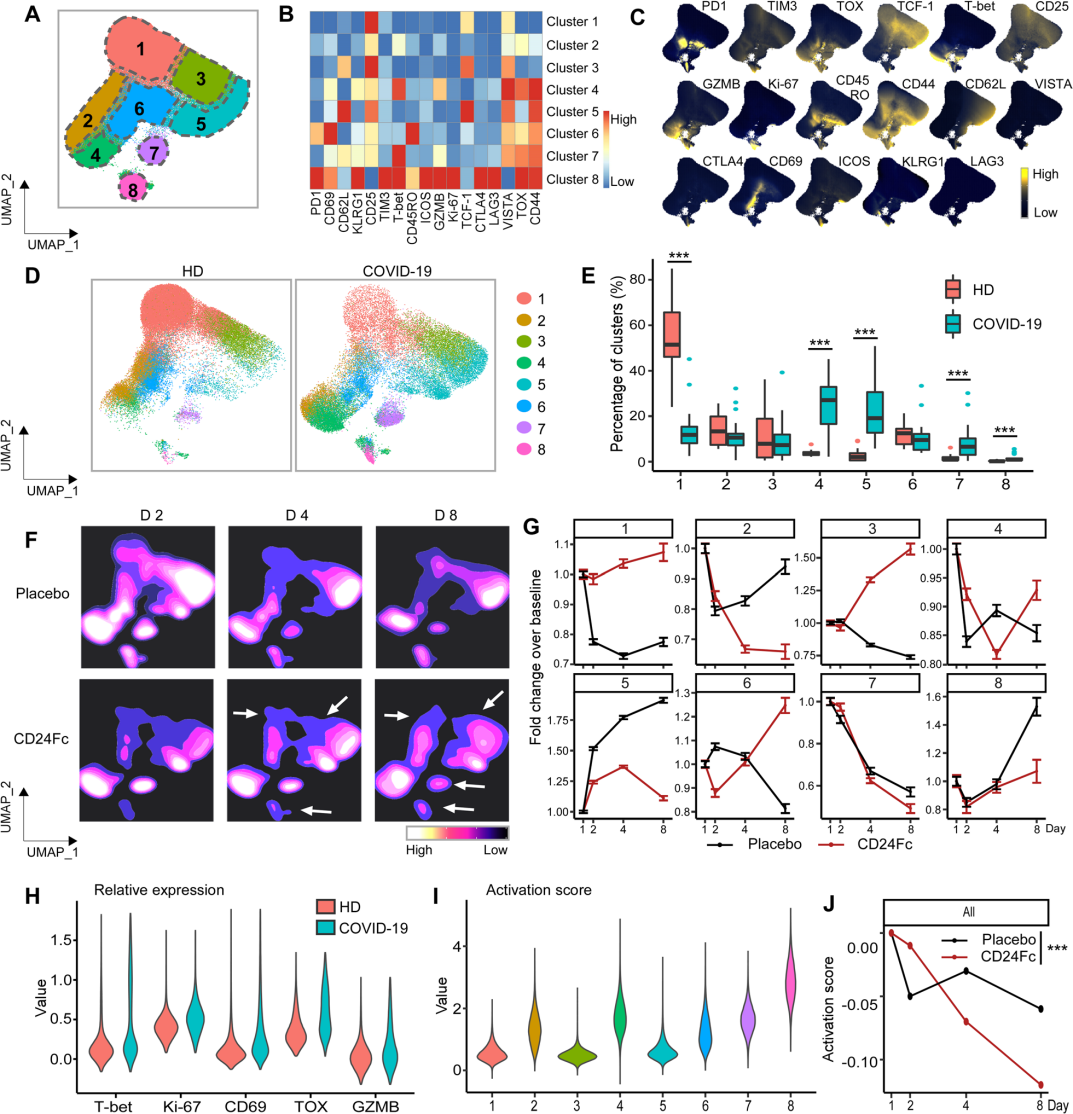
Subcluster analysis of peripheral blood CD8^+^ T cells in COVID-19 patients: activation following SARS-CoV-2 infection is dampened by CD24Fc treatment. 1,466,822 CD8^+^ cells from HD (*n* = 17) and COVID-19 (*n* = 22) patients were clustered using an unbiased multivariate *t*-mixture model, which identified eight statistically distinct CD8^+^ sub-clusters that reflect different activation states. The relative similarity of each cell and cell cluster on the two-dimensional UMAP space were visualized with a 10% downsampling (**A**). Using median expression of flow cytometry markers, a cluster-by-marker heatmap was generated to characterize the subsets (**B**) and visualize individual marker expression patterns on the UMAP space (**C**). To understand the effect of SARS-CoV-2 infection on cell population dynamics, a comparison was made with UMAP plots (**D**) and cluster frequencies (**E**) of HD vs. baseline COVID-19 patient samples (*p* < 0.001 for clusters 1, 4, 5, 7, 8). The samples from COVID-19 patients 2, 4, and 8 days after CD24Fc vs. placebo are displayed using contour plots to represent density of cells throughout regions of the UMAP space (**F**, white arrows indicate visual changes between CD24Fc vs. placebo). Cluster population dynamics as fold change over baseline in each treatment group are shown (**G**; sample distribution described in Fig. 1F; *p* < 0.001 for cluster 1–8). To better characterize the activation status of CD8 T cells, a subset of markers (T-bet, Ki-67, CD69, TOX, GZMB) was linearly transformed to create a univariate cell-level activation score (**H**), where highly activated cell clusters (such as cluster 8) had highest activation scores (**I**). A GLMM was fit to the longitudinal cell-level activation scores to assess the effect of CD24Fc treatment on activation scores over time (**J**). The *p*-values in **E** and **J** were calculated using Wilcoxon rank-sum test and Kenward-Roger method, respectively. ***p < 0.001

We developed a 25-marker flow cytometry panel to examine the intricacies associated with effector cell (NK and CD4^+^/CD8^+^ T cell) activation and differentiation in response to SARS-CoV-2 infection and CD24Fc treatment (Additional file 1: Table S2). Using our unbiased clustering approach, we identified eight distinct clusters within CD8^+^ T cells from COVID-19 and HD samples (Fig. 2A–C). Clusters 1, 3, and 5 showed naïve and memory like signature with TCF-1 and CD62L expression, and cluster 6 showed increased expression of CD45RO. Cluster 4 showed intermediate T-bet and TOX expression indicating transitory state and cluster 8 expressed multiple activation markers including GZMB, suggestive of hyperactivation in this subset. At baseline, COVID-19 samples showed enriched frequency of clusters 4, 5, 7, and 8, which express markers of activation; HD samples were skewed toward cluster 1, which exhibits a naive phenotype (Fig. 2D–E; *p* < 0.001 for all clusters). To analyze the impact of CD24Fc on CD8^+^ T cell activation, we generated UMAP contour plots for each treatment group (Fig. 2F) and analyzed changes to cluster proportions over time (Fig. 2G). CD24Fc treatment correlated with a modest increase in frequency of the phenotypically-naive cluster 1 over time, whereas placebo-treated patients showed marked decline. Conversely, the proportion of cluster 8 cells (a population whose expression pattern is suggestive of highly activated CD8^+^ T cells) were stagnant in CD24Fc-treated patients, compared to the marked increase seen in the placebo group (Fig. 2G).

While tracking cluster proportions over time provides an unbiased global view of the data, these statistically distinct cell clusters may not always correspond perfectly to biologically distinct cell types. Therefore, we augmented the unbiased clustering analysis with a semi-supervised approach to define a CD8^+^ T cell activation score. Known markers of CD8^+^ T cell activation (T-bet, Ki-67, CD69, TOX, and GZMB) were significantly increased in baseline COVID-19 patients compared to HD (Fig. 2H), supporting our hypothesis that SARS-CoV-2 infection increases peripheral T cell activation. To create a unified cell-level activation score, we used PCA to implement dimension reduction of the cell-by-activation marker expression data for all baseline COVID-19 and HD cells. The first principal component (PC1) loadings of each activation marker were used as coefficients in a linear model for defining the activation score (Additional file 1: Table S5). Thus, while we manually selected key T cell activation markers, we determined the relative contribution of each activation marker to the final activation score in a data-adaptive manner, yielding a semi-supervised approach. We observed positive PC1 loadings and positive average log-fold changes for each activation marker, confirming that higher activation scores reflect higher T cell activation (Additional file 1: Table S5). Distributions of activation scores across cell clusters also confirmed that more highly activated cell subsets feature higher activation scores (Fig. 2I). To characterize the effect of CD24Fc treatment on global CD8^+^ T cell activation, we adopted a GLMM of activation scores over time. While CD8^+^ T cell activation scores at baseline were not statistically different between groups, the predicted mean activation scores indicate significantly different trajectories between placebo and CD24Fc groups over time (Fig. 2J; *p* < 0.001). Thus, we conclude that CD24Fc treatment significantly reduced hyperactivation of CD8^+^ T cells compared to placebo.

**Fig. 3:**
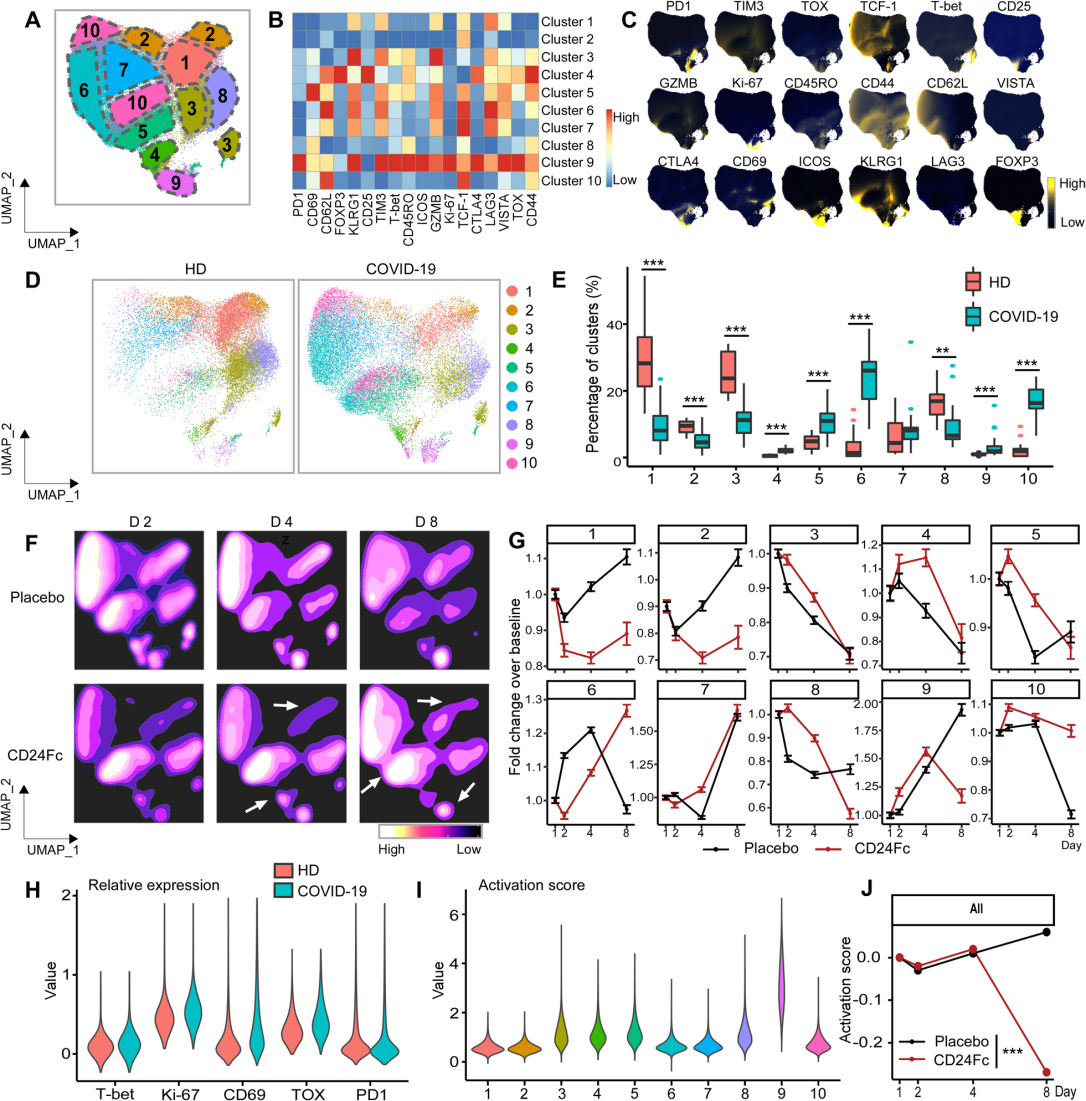
Subcluster analysis of peripheral blood CD4^+^ T cells in COVID-19 patients: activation following SARS-CoV-2 infection is dampened by CD24Fc treatment. We clustered 1,203,034 CD4^+^ cells from HD (*n* = 17) and COVID-19 (*n* = 22) patients using an unbiased multivariate *t*-mixture model, which identified 10 CD4^+^ sub-clusters that reflect statistically distinct cell activation states. We visualized the relative similarity of each cell and cell cluster on the two-dimensional UMAP space with a 10% downsampling (**A**). Using median expression of flow cytometry markers, we generated a cluster-by-marker heatmap to characterize the subsets (**B**) and visualized individual marker expression patterns on the UMAP space (**C**). To understand the effect of SARS-CoV-2 infection on cell population dynamics, we compared UMAP plots (**D**) and cluster frequencies (**E**) of HD vs. baseline COVID-19 patient samples (*p* < 0.001 for clusters 1–6, 9, 10; cluster 8, *p* = 0.002). We visualized samples from COVID-19 patients D2, 4, and 8 after CD24Fc vs. placebo using contour plots to represent the density of cells throughout regions of the UMAP space (**F**). We describe cluster population dynamics as fold change over baseline in each group (**G**; sample distribution described in Fig. 1F; *p* < 0.001 for cluster 1–10). To better characterize the activation status of CD4 T cells, we linearly transformed a subset of markers (T-bet, Ki-67, CD69, TOX, PD1) to create a univariate cell-level activation score (**H**), where highly activated cell clusters (such as cluster 9) had highest activation scores (**I**). We fit a GLMM to our longitudinal cell-level activation scores to assess the effect of CD24Fc treatment on activation scores over time (**J**; *p* < 0.001). The *p*-values in **E** and **J** were calculated using the Wilcoxon rank-sum test and the Kenward–Roger method, respectively. **p < 0.01; ***p < 0.001

**Fig. 4:**
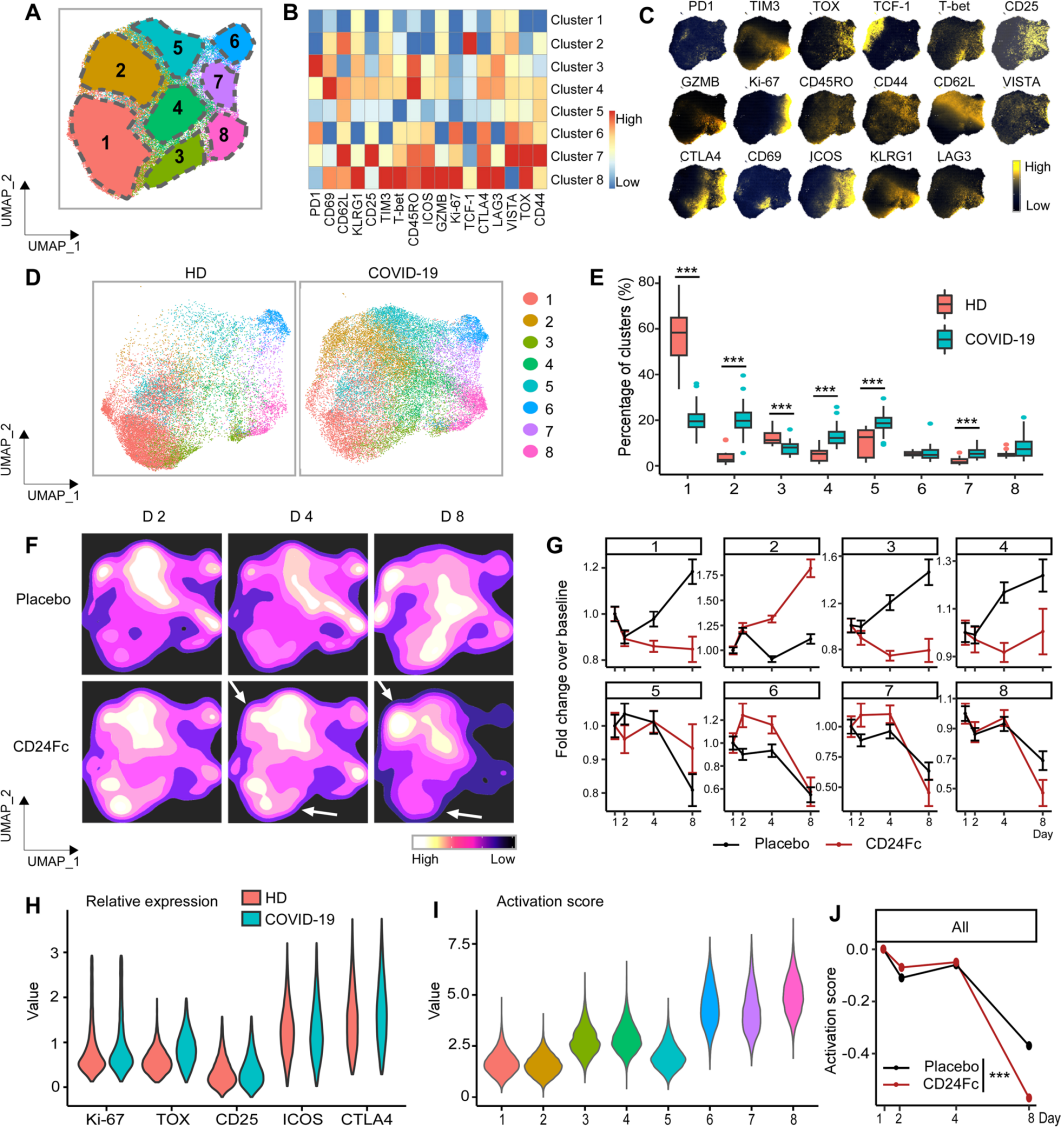
Subcluster analysis of peripheral blood Foxp3^+^ Treg cells in COVID-19 patients: activation following SARS-CoV-2 infection is dampened by CD24Fc treatment. We clustered 98,525 Foxp3^+^ Treg cells from HD (*n* = 17) and COVID-19 (*n* = 22) patients using an unbiased multivariate *t*-mixture model, which identified 8 Foxp3^+^ Treg sub-clusters that reflect statistically distinct cell activation states. We visualized the relative similarity of each cell and cell cluster on the two-dimensional UMAP space with a 10% downsampling (**A**). Using median expression of flow cytometry markers, we generated a cluster-by-marker heatmap to characterize the subsets (**B**) and visualized individual marker expression patterns on the UMAP space (**C**). To understand the effect of SARS-CoV-2 infection on cell population dynamics, we compared UMAP plots and cluster frequencies of HD vs. baseline COVID-19 patient samples (**D** and **E;** *p* < 0.001 for clusters 1–5, 7). We visualized samples from COVID-19 patients D2, 4, and 8 after CD24Fc vs. placebo using contour plots to represent the density of cells throughout regions of the UMAP space (**F**). We describe cluster population dynamics as fold change over baseline in each treatment group (**G**; sample distribution described in Fig. 1F; *p* < 0.001 for cluster 1, 4, 6–8; cluster 3, *p* = 0.004). To better characterize the activation status of Treg cells, we linearly transformed a subset of markers (Ki-67, TOX, CD25, ICOS, CTLA4) to create a univariate cell-level activation score (**H**), where highly activated cell clusters (such as clusters 6, 7, 8) had highest activation scores (**I**). We fit a GLMM to our longitudinal cell-level activation scores to assess the effect of CD24Fc on activation scores over time (**J**). The *p*-values in **E** and **J** were calculated using the Wilcoxon rank-sum test and the Kenward-Roger method, respectively. ***p < 0.001

CD4^+^ T cell activation also plays an important role in the immune response to SARS-CoV-2 infection, so we applied the analysis strategy presented above to this population [35]. To comprehensively understand the role of CD4^+^ T cells and Foxp3^+^ regulatory T cells (Treg), we analyzed total CD4^+^ T cells, including Foxp3^+^ subset (Fig. 3), and then the Foxp3^+^ Tregs exclusively (Fig. 4). We added Foxp3 to the existing 24-marker flow cytometry panel for the identification of Foxp3^+^ Tregs. Using our unbiased clustering approach, we identified 10 clusters of statistically distinct CD4^+^ T cell sub-populations that we projected onto UMAP space to observe global clustering patterns (Fig. 3A, D). To characterize cell clusters in terms of differential marker expression, we computed median expression levels of the 18 markers in the CD4^+^ flow cytometry panel and plotted cell-level marker expression for each marker on the UMAP space (Fig. 3B, C). Clusters 1 and 2 showed lowest CD44 expression indicating naïve-like phenotype, and cluster 3 showed GZMB expression. Cluster 4 showed highest expression level of CD25 and FOXP3 suggestive of Treg. Clusters 5, 6, 7, and 10 expressed intermediate to high levels of TCF1, and Cluster 9 expressed multiple activation markers. CD4^+^ T cells revealed dramatic changes in the relative representation of each cluster upon SARS-CoV-2 infection. Similar to the CD8^+^ T cell activation pattern we observed, CD4^+^ T cells from COVID-19 patients showed a significant reduction in clusters with lower activation marker expression levels, including clusters 1 (*p* < 0.001), 2 (*p* < 0.001), and 8 (*p* = 0.002), and a significant increase in clusters with higher activation marker expression levels, including clusters 4, 5, 6, 9, and 10 (all *p* < 0.001). These results suggest that clusters 1 and 2 are largely composed of less activated CD4^+^ T cells, while other clusters are composed of relatively more activated phenotypes (Fig. 3D, E).

Next, we assessed the short-term longitudinal effect of CD24Fc treatment on CD4^+^ T cells in COVID-19 patients. Using UMAP contour plots to visualize temporal and treatment-level changes in CD4^+^ T cell dynamics (Fig. 3F), we quantified fold-changes in populations over time (Fig. 3G). In contrast to our CD8^+^ T cell results, wherein the phenotypically-naive cluster 1 sustained its level during CD24Fc treatment (Fig. 2), clusters 1 and 2 from the CD4^+^ T cell population decreased upon CD24Fc treatment, which we believe reflects reduced activation. Clusters 4, 5, and 10 were increased by CD24Fc treatment. Cluster 4 showed high expression level of CD25 and FoxP3 (likely Tregs), while clusters 5 and 10 showed intermediate-to-high levels of CD62L and TCF-1 expression. Cluster 9, which expressed multiple activation markers and was presumably composed of hyper-activated cells, was decreased by CD24Fc treatment, similar to CD8^+^ T cell results.

Using the univariate cell-level activation workflow described above, we determined CD4^+^ T cell activation scores. Known markers of CD4^+^ T cell activation (T-bet, Ki-67, CD69, TOX, and PD1) were significantly increased in baseline COVID-19 patients compared to HD (Fig. 3H). Distributions of activation scores across cell clusters also confirmed that more highly activated cell subsets feature higher activation scores (Fig. 3I). Predicted mean activation scores indicate significantly different trajectories between the placebo and CD24Fc groups over time; CD24Fc-treated samples had significantly lower CD4^+^ cell activation levels relative to placebo (overall *p* < 0.001; Fig. 3J). Baseline values for CD4^+^ T cell activation were not statistically different between groups. In contrast, mean activation scores were significantly different between placebo and CD24Fc-treated at all other time points (D2, *p* = 0.001; D4, *p* < 0.001; D8, *p* < 0.001), with the most marked difference on day 8. Thus, we conclude that the attenuation of lymphocyte hyperactivation extends to the CD4^+^ T cell compartment.

We performed the same analyses on Foxp3^+^ Tregs exclusively (Fig. 4) and found that COVID-19 was associated with hyperactivation in this population as well. Cluster 1 showed lowest expression of CD44 suggesting less activated phenotype. Clusters 3 and 4 showed highest expression levels of PD1 and CD69, respectively, and cluster 2 exhibited highest TCF1 expression level. Clusters 6, 7, and 8 expressed multiple activation markers including CTLA4. Upon SARS-CoV-2 infection, clusters 1 and 3, which represent less activated phenotype, were downregulated and clusters 6, 7, and 8 reflecting more activated phenotype increased when COVID-19 patient samples were compared with HD samples. CD24Fc treatment was associated with a substantial reduction by day 8 (Fig. 4G) in the proportion of cells belonging to the most hyperactivated cell cluster (Treg cluster 8; Fig. 4I). Using the GLMM activation score model, we found a significant reduction in Foxp3^+^ Treg activation associated with CD24Fc treatment by day 8 (*p* < 0.001), while we failed to detect a significant difference in predicted activation scores between treatment groups at earlier time points (Fig. 4J).

### CD24Fc reduces NK cell dysregulation

**Fig. 5:**
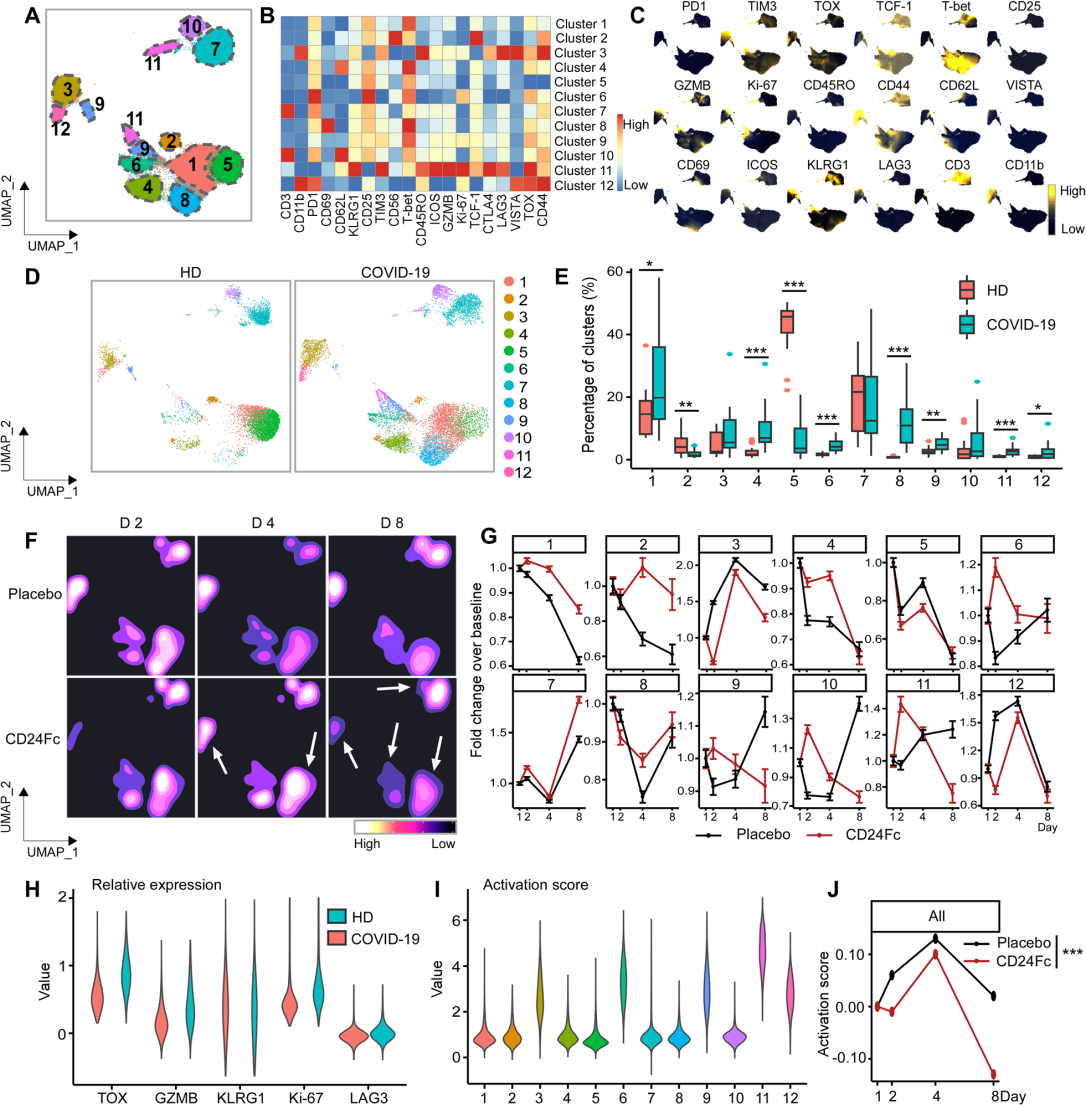
Subcluster analysis of peripheral blood NK cells in COVID-19 patients: activation following SARS-CoV-2 infection is dampened by CD24Fc treatment. CD56^+^ cells (*n* = 783,623) from HD (*n* = 17) and COVID-19 (*n* = 22) patients were clustered using an unbiased multivariate *t*-mixture model, which identified 12 sub-clusters that reflect statistically distinct CD56^+^ T cell activation states. The relative similarity of each cell and cell cluster on the two-dimensional UMAP space were visualized with a 10% downsampling (**A**). Using median expression of flow cytometry markers, a cluster-by-marker heatmap was generated to characterize subsets (**B**) and visualize individual marker expression patterns on UMAP space (**C**). To understand effect of SARS-CoV-2 infection on NK population dynamics, we compared UMAP plots (**D**) and cluster frequencies (**E**; cluster 1, *p* = 0.04; cluster 2, *p* = 0.003; cluster 9, *p* = 0.002; cluster 12, *p* = 0.03; *p* < 0.01 for clusters 4–6, 8 and 11) of HD vs. baseline COVID-19 samples. D2, 4, 8 samples from placebo and CD24Fc-treated groups were visualized using contour plots to represent density of cells throughout regions of the UMAP space (**F**, white arrows indicate visual changes between CD24Fc vs. placebo). The cluster population dynamics as fold change over baseline in each group was shown (**G**; sample distribution described in Fig. 1; *p* < 0.001 for cluster 1, 3–12). To better characterize the activation status of NK cells, a subset of markers (TOX, GZMB, KLRG1, Ki-67, LAG-3) was linearly transformed to create a univariate cell-level activation score (**H**), where highly activated cell clusters (such as cluster 11) had highest activation scores (**I**). A GLMM was fit to the longitudinal cell-level activation scores to assess the effect of CD24Fc on activation scores over time (**J**). The *p* values in **E** and **J** were calculated using Wilcoxon rank-sum and Kenward–Roger method, respectively. *p < 0.05; **p < 0.01; ***p < 0.001

The increased number of NK cells in samples from patients with COVID-19 (Fig. 1C, D, cluster 10) implies they play an important role in SARS-CoV-2 infection. We investigated the activation and functional status of NK cells using our unbiased clustering and visualization approach and identified 12 statistically distinct NK cell clusters, which we visualized on heatmaps and UMAPs (Fig. 5A–C). Clusters 7 and 10 showed CD3 expression indicating NKT cell property, and cluster 11 expressed multiple activation markers suggesting hyperactivated phenotype. Clusters 1 and 5 showed minimal expression of activation markers indicative of resting NK cells, and clusters 3 and 12 showed CD11b expression. Cluster 5, the most highly represented cluster in HD samples, displayed an expression pattern suggestive of a less activated population; it was significantly downregulated in COVID-19 patients (*p* < 0.001; Fig. 5D–E). Samples from COVID-19 patients also revealed a significant reduction in cluster 2 (*p* = 0.003) and expansion of clusters 1, 4, 6, 8, 9, 11, and 12 (Fig. 5D–E; *p* = 0.04 for cluster 1; *p* = 0.002 for cluster 9; *p* = 0.03 for cluster 12; *p* < 0.001 for clusters 4, 6, 8, 11).

To understand the role of CD24Fc treatment on NK cell population dynamics, we generated UMAP contour plots to visualize temporal and treatment-based changes (Fig. 5F), and quantified these differences (Fig. 5G). Clusters 1 and 2, which showed a more naive phenotype, were increased by CD24Fc, whereas cluster 11, which expresses multiple activation markers, was decreased. To visualize activation, known NK cell activation markers (TOX, GZMB, KLRG1, Ki-67, and LAG3) were assessed (Fig. 5H) and plotted per cluster (Fig. 5I). Using a GLMM of activation scores over time, we found that while baseline values for NK cell activation were not statistically different, the mean activation scores were significantly different between placebo and CD24Fc groups throughout the study duration (*p* < 0.001; Fig. 5J). Thus, CD24Fc treatment rapidly normalized NK cell activation status, and the impact was sustained throughout the study period.

### CD24Fc attenuates systemic cytokine response

**Fig. 6:**
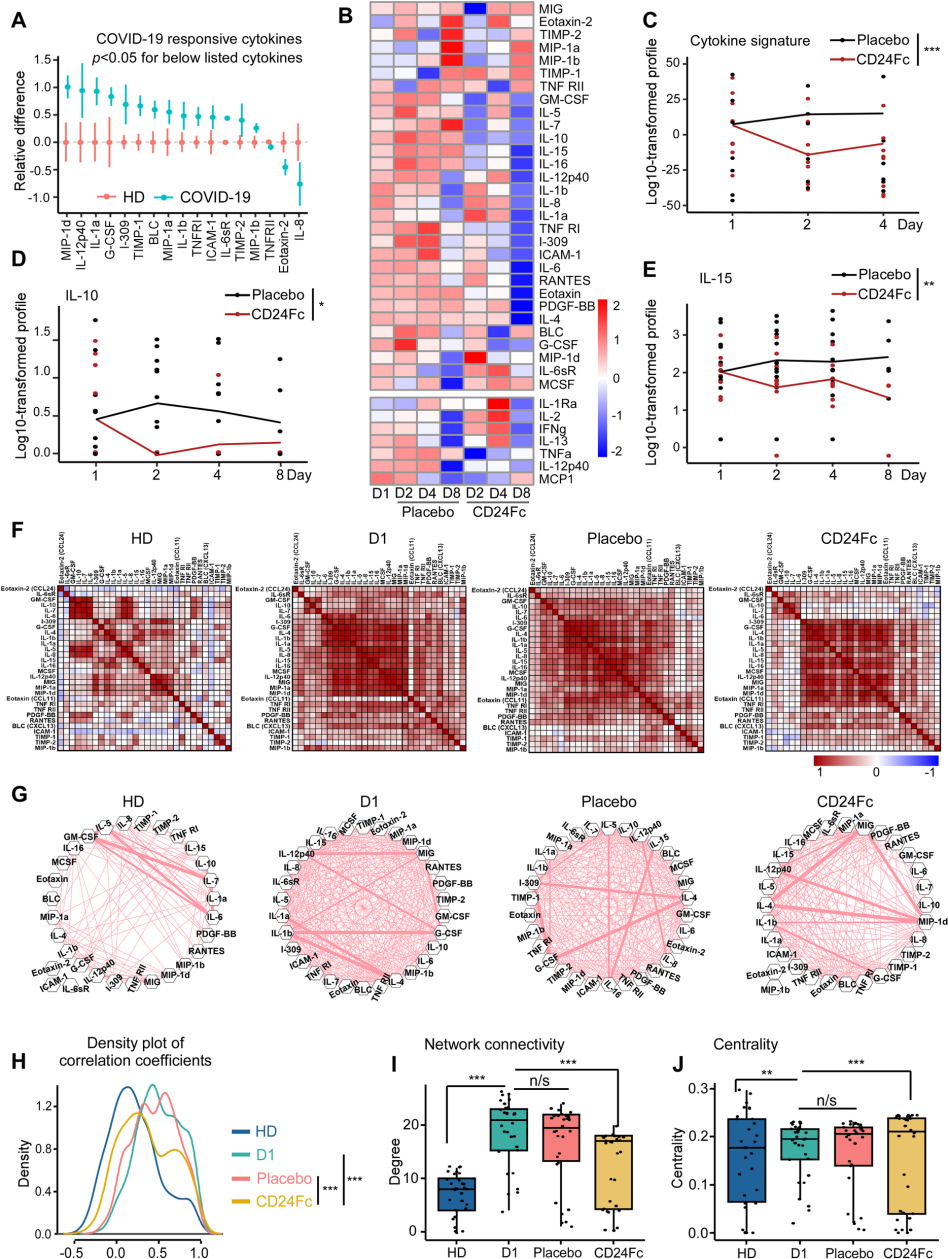
CD24Fc treatment downregulates systemic cytokine response in patients with COVID-19. Relative differences in plasma concentrations of cytokines/chemokines between HD (*n* = 25) and COVID-19 patients (*n* = 22) is shown. Values were log-transformed and evaluated using independent sample t-test. Significantly up- and down-regulated markers are shown (**A**). Heatmap analysis (**B**) visualized relative levels of cytokines/chemokines (Placebo: D1 *n* = 12, D2 *n* = 12, D4 *n* = 11, D8 *n* = 5; CD24Fc: D1 *n* = 10, D2 *n* = 10, D4 *n* = 9, D8 *n* = 3). Using log-10 transformation of cytokine concentrations (dots) and GLMM-predicted fixed effects trends (lines), changes in IL-10 (**C**; p = 0.05) and IL-15 (**D**; p = 0.002) in CD24Fc (red) and placebo (black) groups were revealed. Values and trend lines were centered at D1 mean. *p*-value was calculated using the Kenward–Roger method. The cytokine score was analyzed longitudinally using weighed sum approach (**E**; *p* < 0.001). Using Pearson correlation matrices (**F**) and network maps (**G**; weight of edge represents correlation coefficient), 30 plasma markers in HD (*n* = 25), COVID-19 baseline (D1, *n* = 22), placebo (pooled D2–D8, *n* = 28), and CD24Fc-treated (pooled D2–D8, *n* = 24) groups were visualized. Using these correlation coefficients, a density plot (**H**; D1 vs placebo, *p* = 0.07; D1 vs CD24Fc, *p* < 0.001; placebo vs CD24Fc, *p* < 0.001) was constructed. Kolmogorov–Smirnov test was used to evaluate equality of densities between groups. Analysis of connectivity (**I**) and centrality analysis of cytokine network (**J**) display the cytokine expression relationships. Network connectivity plots display highly correlated connections for each cytokine (i.e., node degree) and was evaluated using paired *t*-test. Centrality analysis of cytokine network used eigenvector centrality score that considers global network connectivity and correlation coefficients between cytokines (HD vs D1, *p* < 0.001; D1 vs placebo, *p* = 0.08; D1 vs CD24Fc, *p* < 0.001). Bartlett’s test evaluated the significance of variance of centrality scores (HD vs D1, *p* = 0.013; D1 vs placebo, *p* = 0.17; D1 vs CD24Fc, *p* = 0.008). Each dot in **I** and **J** represents a cytokine. *p < 0.05; **p < 0.01; ***p < 0.001

The profound changes in lymphocyte dynamics after CD24Fc treatment indicate that CD24Fc exerts its effect by regulating the systemic cytokine levels. To test this hypothesis, we compared plasma cytokine concentrations from HD and COVID-19 patients treated with CD24Fc or placebo. We used multiplex ELISA and Luminex analysis platforms to test 37 cytokines in total. Fifteen out of 37 tested cytokines were significantly elevated (*p* < 0.05) during SARS-CoV-2 infection (*p* < 0.05, Fig. 6A, Additional file 1: Fig. S2A-B). These included cytokines associated with type 1 (IL-12p40, CXCL9, IL-15) and type 3 (IL-1α, IL-1β, RANTES) immunity, and chemokine MCP-1 (CCL2) that recruits monocytes and T cells to the sites of inflammation. Only three of 37 cytokines, including TNRFII, Eotaxin-2 and IL-8 were significantly downregulated in COVID-19 patients (*p* < 0.05; Fig. 6A).

We next studied the impact of CD24Fc on cytokine expression in patients with COVID-19. As shown in Fig. 6B, several cytokines (GM-CSF, IL-5, IL-7, IL-10) and chemokines (MIG, MIP-1α, MIP-1β) were down-modulated over time. Serum levels dynamics of selected individual cytokines are shown in Fig. 6C, D and Fig. S2C. At one week after treatment initiation, many cytokines and chemokines were reduced by tenfold or more. Notably, many of these inflammatory proteins were selectively reduced in the CD24Fc-treated patients or downregulated more rapidly compared to placebo-treated patients. Specifically, CD24Fc significantly down-modulated plasma levels of IL-10 and IL-15 (*p* = 0.05 and *p* = 0.002, respectively; Fig. 6C, D). Other tested cytokines implicated in COVID-19 pathogenesis, including IL-6 and GM-CSF [39], exhibit a similar trend toward a selective downregulation by CD24Fc, albeit these did not reach the levels of statistical significance (Fig. 6B, Additional file 1: Fig S2C). To increase the statistical power of the analysis of the influence of CD24Fc on systemic cytokine response, we calculated a cytokine scores for each treatment group by integrating expression of all markers tested by multiplex ELISA platform using weighted sum approach. Analysis of cytokine scores demonstrated a significant decrease in CD24Fc-treated groups compared to placebo (*p* < 0.001; Fig. 6E). This finding was independently confirmed using Autoencoder [28] and PCA (Additional file 1: Fig. S2D).

To better understand the global modulation of systemic cytokine response by CD24Fc treatment, we studied correlations between individual cytokines across groups. Correlation matrices wherein darker red lines indicate stronger correlation (Fig. 6F) showed that only a few groups of cytokines were co-expressed by HD. However, the numbers of co-regulated cytokines dramatically increased in baseline COVID-19 samples (vs. HD controls) indicating activation of coordinated cytokine response. Remarkably, samples from CD24Fc-treated patients (pooled over time) showed a decline in cytokine correlations compared to baseline or placebo treatment. Similarly, cytokine network plots connecting cytokines with moderate and strong associations (Pearson correlation r > 0.4 [29]) showed lower overall interconnectedness in CD24Fc group as compared to baseline or placebo treatment (right two panels, Fig. 6G). The overall cytokine network correlations and connectivity in CD24Fc-treated patients were significantly different from baseline or placebo treatment (Fig. 6H, I; *p* < 0.001 for both).

**Fig. 7:**
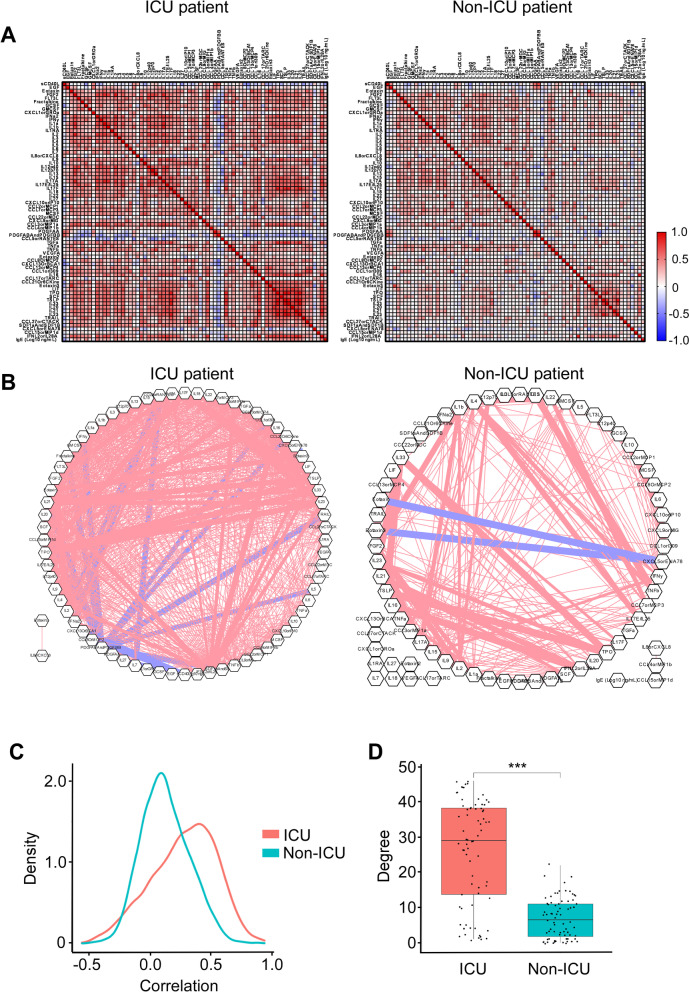
Patients with severe COVID-19 that require an ICU treatment display increased correlation and connectivity of the systemic cytokine network. We analyzed correlation (**A**) and connectivity (**B**) between circulating cytokines and chemokines in COVID-19 patients that either required (ICU patients), or did not require an ICU treatment (non-ICU patients). Cytokine measurements were obtained from previously published dataset [40]. Analysis was performed as described in Fig. 6. A density plot constructed based on connectivity between plasma cytokines is shown in **C**. **D** shows an association between the severity of COVID-19 infection and the degree of the connectivity between plasma cytokines with severe UCU cases displaying higher degree of connectivity. *p*-value was calculated using Wilcoxon Rank sum

To understand the relevance of decreased correlation and connectivity of the cytokine network in CD24Fc-treated patients to disease severity and therapeutic effect, we analyzed a previously published dataset of cytokine expression in serum from patients with COVID-19 that were either treated in the intensive care unit (ICU patients) or did not require ICU treatment (non-ICU patients) [40]. Notably, we found that inter-cytokine correlation and connectivity were lower in non-ICU patients than ICU patients (Fig. 7). These data suggest that the increased blood cytokine network correlation and connectivity analysis we developed are associated with increased COVID-19 disease severity, while mild disease (without the need for ICU treatment) is characterized by lower correlation and connectivity. Therefore, decreased correlation and connectivity of the cytokine network is likely a novel useful tool to examine the therapeutic efficacy of anti-inflammatory agents.

To identify factors that may play an important role in response to CD24Fc, we calculated centrality scores [32] for individual cytokines based on their connectivity and correlations within the global cytokine network (Additional file 1: Table S6). The variances of the centrality scores of 30 cytokines were lower in baseline and placebo-treated COVID-19 patients compared to HD and CD24Fc-treated COVID-19 patients (Fig. 6J). These data indicate that distinct cytokines are highly heterogeneous in terms of their interconnectedness with other cytokines (centrality) in healthy individuals. Upon SARS-CoV-2 infection, cytokine centralities become more uniform, and subsequent CD24Fc treatment abrogates this effect (Fig. 6J).

## Discussion

Patients enrolled in the phase III SAC-COVID clinical trial, a subpopulation of which were studied herein, demonstrated accelerated clinical recovery following CD24Fc treatment compared to placebo. CD24Fc was generally well-tolerated, reduced disease progression, and shortened hospital length of stay (results under review in Welker et al*.* “Therapeutic Efficacy and Safety of CD24Fc in Hospitalized Patients with COVID-19,” submitted to *Lancet Infectious Diseases*). Given the proposed mechanism of action and pathophysiology of SARS-CoV-2, we hypothesized that CD24Fc reduced the hyperactive systemic immune responses in infected patients leading to an accelerated return to immune homeostasis. Using deep immune profiling of longitudinal samples combined with our in-depth bioinformatic analysis, we uncovered the effects of CD24Fc on the systemic host immune response. Overall, we found that CD24Fc treatment blunted immune cell activation across several compartments including B cells, CD4^+^ T cells CD8^+^ T cells and NK cells, and facilitated the return to a more normal phenotype following SARS-CoV-2 infection.

Comparing baseline COVID-19 patients with HD allowed us to identify the immune cell populations driving pathogenesis. As expected, we saw a significant increase in activated CD8^+^ T and NK cells in SARS-CoV-2-infected patients. We augmented the unbiased clustering analysis with a semi-supervised approach to defining an unbiased activation score. CD24Fc-treated patients demonstrated a significant reduction in activation score over time for CD8^+^ T, CD4^+^ T, and NK cells compared to placebo-treated patients.

The changes in overall immune cell population dynamics between HD and COVID-19 patients are intriguing and offer two separate interpretations. CD24Fc may preferentially block the differentiation of mature B cells into effector plasma cells, resulting in relatively fewer plasma B cells (cluster 6) and more mature B cells (cluster 8). Alternatively, CD24Fc treatment may reduce the systemic burden of SARS-CoV-2 infection, which would limit the number of plasma cells due to accelerated recovery. In either scenario, the correlation between decreased circulating plasma cells in CD24Fc-treated patient samples suggests significant immuno-modulatory roles of this treatment. The ability of patients to mount an effective anti-Spike antibody response was not compromised by CD24Fc treatment.

An aberrant and rapid increase in a broad spectrum of pro-inflammatory cytokines, known as a cytokine storm, plays a central role in the pathogenesis of ARDS and other severe complications of SARS-CoV-2 infection [41]. Of note, antibodies targeting IL-6R can effectively treat the cytokine storm associated with immunotherapy and many chronic inflammatory diseases [42]. However, the results of several clinical trials that investigated the benefits of IL-6 antagonists in patients with COVID-19 were not consistent [43, 44]. Although the meta-analysis of 27 randomized clinical trials including 19,930 COVID-19 patients in total did show lower 28-day mortality with IL-6 antagonists administration [45], high variability of the treatment outcome suggests that broader-acting interventions against COVID-19-associated cytokine storm may be needed to achieve a reliable benefit. Our longitudinal analysis revealed a broad-spectrum up-regulation of systemic cytokines in patients with severe COVID-19. More importantly, CD24Fc treatment caused rapid and sustained reduction of the systemic cytokine response as indicated by down-modulation of the combined cytokine score, which was derived using expression levels of 30 cytokines and chemokines. This broad effect may explain the significant therapeutic efficacy of CD24Fc in treating hospitalized COVID-19 patients.

In addition, we identified two cytokines that were significantly downregulated after CD24Fc treatment: IL-10 and IL-15. Both are linked with COVID-19 severity, increased intensive care admission, and COVID-19-associated death [46–48]. Although generally associated with immunosuppressive functions, IL-10 can also stimulate NK and CD8^+^ T cells and induce B cell proliferation and antibody production [49]. IL-15 promotes activation and expansion of NK and CD8^+^ T cells [50, 51]. Thus, CD24Fc may prevent pathological activation of NK and CD8^+^ T cells by suppressing IL-10 and IL-15 production. Since IL-15 also promotes activation and recruitment of neutrophils to site of inflammation, CD24Fc may suppress COVID-19-associated neutrophil activation and neutrophilia [52]. Furthermore, CD24Fc may limit viral replication by suppressing IL-10 production, which has been shown to enhance viral replication of HIV, HCV and HBV [53]. These ideas warrant further investigation.

Importantly, unlike HD, COVID-19 patients displayed strong positive correlations between inflammatory cytokines, consistent with broad misfiring of host immune responses [38, 40, 54]. Notably, CD24Fc treatment reduced systemic cytokine levels and diminished correlations and connectivity in SARS-CoV-2-infected individuals, thus reshaping the systemic cytokine network toward a less tightly co-regulated state characteristic of homeostasis. Based on an analysis of the global cytokine landscape, we conclude that CD24Fc mitigates the exacerbated host systemic inflammatory responses to SARS-CoV-2. This conclusion was corroborated by the decrease of cytokine correlation and connectivity in patients with mild COVID-19 infections as compared to patients with severe disease that required an ICU treatment that we uncovered using a published COVID-19 patient dataset [40]. In addition, a detailed investigation of individual inflammatory markers revealed potential mechanisms of COVID-19 severity reduction by CD24Fc. Of note, our network-based analysis to demonstrate connectivity and centrality of multiple inflammatory mediators simultaneously may prove useful for the study of other disease settings, especially in the realm of developing and monitoring the impact of anti-inflammatory therapeutics.

## Conclusions

In conclusion, the data presented here offer unique immunological insights that underscore the encouraging clinical findings from the SAC-COVID trial. These results strongly support further investigation of CD24Fc for various inflammatory conditions, including COVID-19. Indeed, CD24Fc has been tested in Phase II clinical trial to attenuate graft versus host diseases and showed promising efficacy, opening potential use of this drug in other immune related diseases. Our unique cytokine centrality analysis and cellular activation index warrant further study as a prognostic tool for guiding therapy in COVID-19 and other systemic inflammatory conditions.

## Supplementary Information

**Additional file 1**. Supplemental Materials, including list of investigators and affiliations; supplementary figures S1 and S2, and supplementary tables S1, S2, S5 and S6.

**Additional file 2**.** Table S3**. Cytokine concentrations from plasma samples, as measured by multiplex ELISA.

**Additional file 3**.** Table S3**. Cytokine concentrations from plasma samples, as measured by Luminex.

## Data Availability

The datasets used and/or analyzed during the current study are available from the corresponding author on reasonable request. Cytokine data generated during this study are included in this published article and its supplementary information files. Data used for supplementary figure S3 are available at https://doi.org/10.1038/s41586-020-2588-y.

## Abbreviations

ACE2: Angiotensin-converting enzyme 2
ARDS: Acute respiratory distress syndrome
CD24Fc: Soluble CD24
COVID-19: Coronavirus disease 2019
DAMP: Damage-associated molecular patterns
EC: Eigenvector centrality
ELISA: Enzyme-linked immunoassay
Fc: Fragment crystallizable
GLMM: Generalized linear mixed model
Gluc: Gaussia luciferase
GWAS: Genome-wide association study
GZMB: Granzyme B
HBV: Hepatitis B virus
HCV: Hepatitis C virus
HD: Healthy donor
HIV: Human immunodeficiency virus
HMGB1: High-mobility group box 1
IFN: Interferon
IgG1: Immunoglobulin G1
IL: Interleukin
IV: Intravenous
KLRG1: Killer cell lectin-like receptor G1
LAG3: Lymphocyte-activation gene 3
MCP: Monocyte chemoattractant protein
mRNA: Messenger RNA
NF-kB: Nuclear Factor kappa-light-chain-enhancer of activated B cells
NK cells: Natural Killer cells
PBMC: Peripheral blood mononuclear cell
PCA: Principal component analysis
PCR: Polymerase chain reaction
PD1: Programmed cell death protein 1
SARS-CoV-2: Severe Acute Respiratory Syndrome Coronavirus 2
SHP1: Src homology 2 domain-containing protein tyrosine phosphatase 1
Siglec10: Sialic acid-binding Ig-like lectin 10
T-bet: T-box expressed in T cells
TNF: Tumor necrosis factor
TOX: Thymocyte selection-associated HMG BOX
Treg: Regulatory T cells
UMAP: Uniform Manifold Approximation and Projection
