# Supplementary material for: Treatment with soluble CD24 attenuates COVID-19-associated systemic immunopathology
Source: J Hematol Oncol. 2022 Jan 10;15:5. doi: 10.1186/s13045-021-01222-y (PMC8744064; doi:10.1186/s13045-021-01222-y)
Supplement: Supplementary file 1 — Additional file 1. Supplemental Materials, including list of investigators and affiliations; supplementary figures S1 and S2, and supplementary tables S1, S2, S5 and S6. [file 13045_2021_1222_MOESM1_ESM.docx]

**SUPPLEMENTAL MATERIAL**

This appendix has been provided by the authors to give readers additional information about their work.

# LIST OF INVESTIGATORS

**Clinical Trial Site:** The Ohio State University Wexner Medical Center 410 W. Tenth Ave, Columbus OH 43210

**Clinical Laboratory Facility:** OSUCCC Pelotonia Institute for Immuno-Oncology, 460 W. 12th Ave Room 580 Biomedical Research Tower, Columbus OH 43210

**Clinical Investigators:** Carlos Diego Malvestutto, M.D. M.P.H; Zeinab El Boghdadly, M.D.; Mohammad Mahdee Sobhanie, M.D.; Jose Bazan, D.O.; Mark Lustberg, M.D. Ph.D.; Susan Koletar, M.D.; Zihai Li, M.D. Ph.D.; Kelsi Reynolds; Karthik Chakravathy

**Complete list of contributors (alphabetical):** Carter Allen^1,3,4^, Jose Bazan^2^, Chelsea Bolyard^3^, Zeinab El Boghdadly^2^, Donna Bucci^3^, Karthik B. Chakravarthy^3,11^, Yuzhou Chang^1,3,4^, Dongjun Chung^3,4^, Martin Devenport^13^, John P. Evans^12^, Manuja Gunasena^8,9^, Aastha Khatiwada^5^, Susan Koletar^2^, Amrendra Kumar^6,10^, Anqi Li^1,3,11^, Zihai Li^2,3^, Shan-Lu Liu^12^, Yang Liu^13^, Namal P. M. Liyanage^8,9^, Mark Lustberg^2^, Anjun Ma^4^, Qin Ma^4^, Carlos D. Malvestutto^2^, Kelsi Reynolds^3^, Brian P. Riesenberg^3^, Mohammad Mahdee Sobhanie^2^, No-Joon Song^3^, Zequn Sun^5^, Maria Velegraki^3^, Anna E. Vilgelm^3,6,10^, Kevin P. Weller^3^, Menglin Xu^2^, Mohamed Yusuf^6^, Cong Zeng^12^, Pan Zheng^13^.

**Affiliations:**

^1^The Ohio State University, Columbus, OH 43210, USA

^2^Department of Internal Medicine, The Ohio State University College of Medicine, Columbus, OH

^3^The Pelotonia Institute for Immuno-Oncology, The Ohio State University Comprehensive Cancer Center, Columbus, OH 43210, USA

^4^Department of Biomedical Informatics, The Ohio State University College of Medicine, Columbus, OH

^5^Department of Public Health Sciences, Medical University of South Carolina, Charleston, SC

^6^The Ohio State University Comprehensive Cancer Center, Columbus, OH 43210, USA

^7^Department of Microbiology, The Ohio State University College of Arts and Sciences, Columbus, OH 43210, USA

^8^Department of Microbial Infection and Immunity, The Ohio State University College of Medicine, Columbus, OH 43210, USA

^9^Department of Veterinary Biosciences, The Ohio State University College of Veterinary Medicine, Columbus, OH 43210, USA

^10^Department of Pathology, The Ohio State University College of Medicine, Columbus, OH

^11^The Ohio State University College of Medicine, Columbus, OH 43210, USA

^12^Center for Retrovirus Research and Department of Veterinary Biosciences, The Ohio State University, Columbus, OH 43210, USA

^13^OncoC4, Rockville, MD, USA

# SUPPLEMENTARY FIGURES


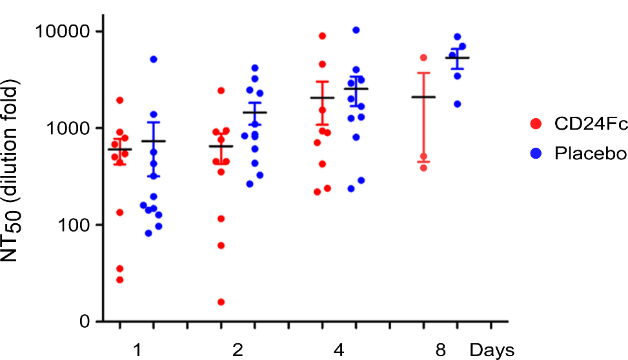


**Figure S1. Comparison of neutralizing antibody against SARS-CoV-2 spike in CD24Fc treated patients and placebo group.** Using our secreted nano luciferase-bearing pseudotyped lentivirus virus neutralization assay, we assessed the neutralizing antibody (nAb) titers for the CD24Fc treated and placebo groups throughout their treatment period. The average 50% neutralization titer (NT_50_) for both groups show an increase in antibody titers from day 0 to day 15, but no significant differences were observed when CD24Fc group were compared to placebo group.

**
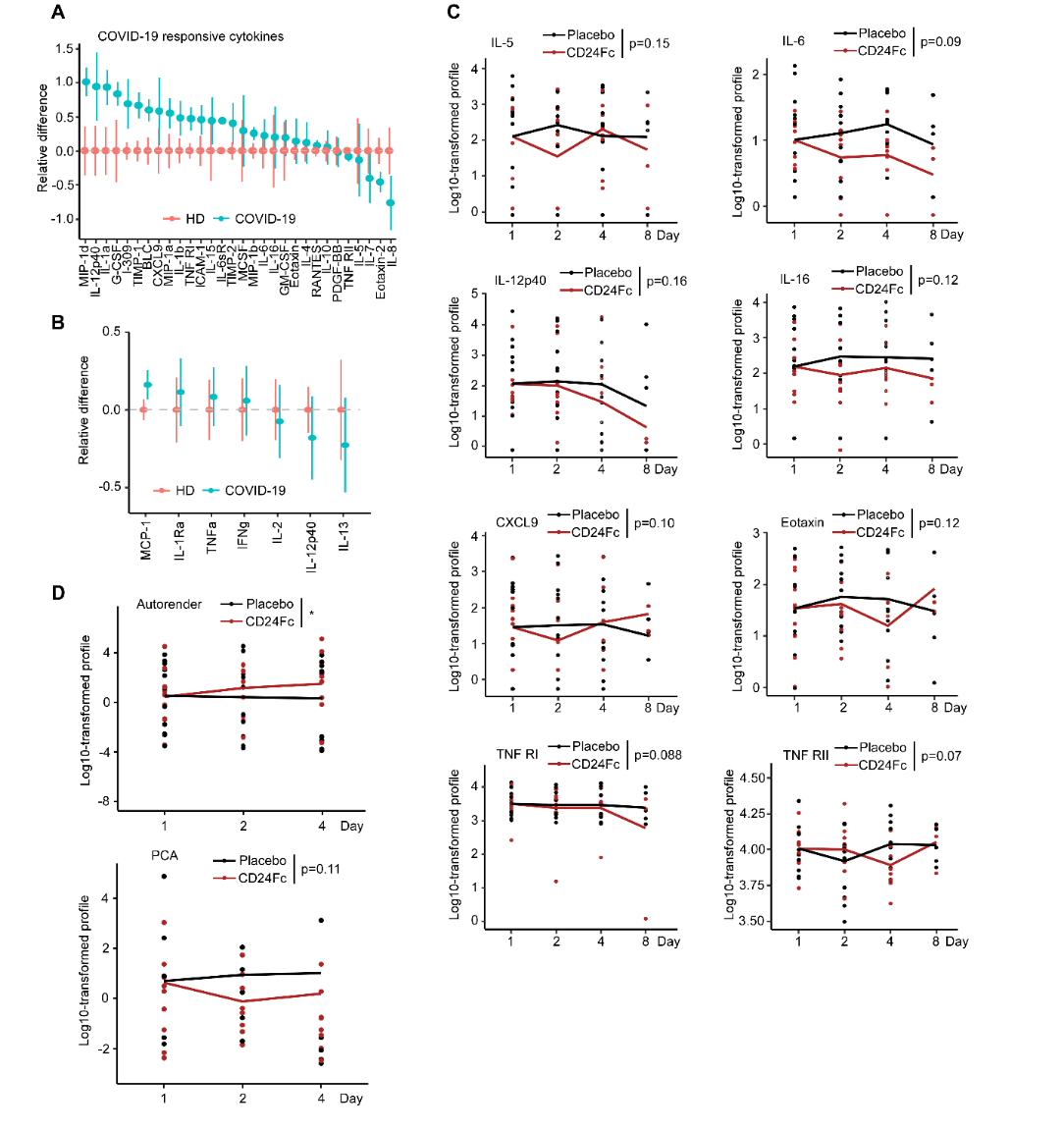
**

**Figure S2. CD24Fc treatment downregulates systemic cytokines response in patients with COVID-19.** We studied plasma cytokine and chemokine levels in HD and COVID-19 patients. Cytokine/chemokine measurements were log-transformed, and relative differences in cytokines in COVID-19 (n=22) compared to HD (n=25) samples were depicted (**Panel A and B**). Graph in Panel A shows data obtained using multiplex-ELISA platform, while graph in panel B presents data of cytokines measured by the Luminex analysis. Independent sample t-test was used to evaluate equality of average cytokine/chemokine levels. A number of other markers displayed trends towards decline in CD24Fc cohort compare to placebo, although these changes were not statistically significant (**Panel C**). Log-10 transformed cytokine measurement (dots) and GLMM predicted fixed effects trends (lines) of IL-5, IL-6, IL-12p40, IL-16, CXCL9, Eotaxin, TNF R1 and TNF RII plasma concentrations in CD24Fc (red) and placebo (black) groups are displayed. The observed values and trend lines are centered at D1 mean. Longitudinal analysis of cytokine score was confirmed using both Autoencoder and PCA approaches (**Panel D**). We applied PCA and autoencoder on the base 10 log-transformed, centered and scaled cytokine data, and investigated the first two principal components (PCs) from the PCA and the three latent components from the autoencoder as cytokine scores. The autoencoder analysis was implemented using the Keras package. Specifically, we set one hidden layer for encoder and decoder, respectively, and three-dimensional embedding as latent layer output. All parameters were trained based on a 3-fold cross-validation. Due to missing data on D8, only D1, D2, and D4 data were used for the cytokine score calculation. For **Panels C** and **D**, the overall differences in trends between CD24Fc and placebo groups across all the time points were evaluated using a GLMM of each measurement. The p-value for evaluating the overall difference in trends between CD24Fc and placebo groups across all the time points was calculated using the Kenward-Roger method.

# SUPPLEMENTARY TABLES

**Table S1. Patient Characteristics.**

| **CHARACTERISTIC** | | **OVERALL** | **PLACEBO** | **CD24Fc** | **P-value** |
| --- | --- | --- | --- | --- | --- |
| **a) Demographics** | | **(N=22)** | **(N=12)** | **(N=10)** |  |
| BMI - median (IQR) | | 31.65 (28.18-38.83) | 31.65 (29.41-39.52) | 32.2 (28.1-37.88) | 0.644 |
| Age, yr - median (IQR) | | 57 (50.25-74.75) | 60.5 (53.5-75) | 55 (49.5-62) | 0.62 |
|  | <65 yr - no. (%) | 15 (68.2) | 7 (58.3) | 8 (80.0) |  |
|  | ≥65 yr - no. (%) | 7 (31.8) | 5 (41.7) | 2 (20.0) |  |
| Sex - no. (%) | |  |  |  | 0.903 |
|  | Female | 8 (36.4) | 5 (41.7) | 3 (30.0) |  |
|  | Male | 14 (63.6) | 7 (58.3) | 7 (70.0) |  |
| Race - no. (%) | |  |  |  | 0.528 |
|  | White | 16 (72.7) | 9 (75.0) | 7 (70.0) |  |
|  | Black/AA | 5 (22.7) | 2 (16.7) | 3 (30.0) |  |
|  | Not Specified | 1 (4.5) | 1 (8.3) | 0 (0.0) |  |
| Ethnicity - no. (%) | |  |  |  | 0.724 |
|  | Hispanic | 4 (18.2) | 3 (25.0) | 1 (10.0) |  |
|  | Non-Hispanic | 18 (81.8) | 9 (75.0) | 9 (90.0) |  |
| Smoking Hx - no. (%) | | 7 (31.8) | 3 (25.0) | 4 (40.0) | 0.77 |
| **b) Co-existing Conditions** | | | | |  |
| Comorbidities - no. (%) | |  |  |  |  |
|  | Obesity (BMI ≥30) | 12 (54.5) | 7 (58.3) | 5 (50.0) | 1 |
|  | Hypertension | 11 (50.0) | 5 (41.7) | 6 (60.0) | 0.669 |
|  | Hyperlipidemia | 8 (36.4) | 5 (41.7) | 3 (30.0) | 0.903 |
|  | Heart Disease | 7 (31.8) | 2 (16.7) | 5 (50.0) | 0.226 |
|  | Diabetes | 7 (31.8) | 3 (25.0) | 4 (40.0) | 0.77 |
|  | Autoimmune Condition | 3 (13.6) | 3 (25.0) | 0 (0.0) | 0.281 |
|  | Cancer | 2 (9.1) | 1 (8.3) | 1 (10.0) | 1 |
|  | HIV | 1 (4.5) | 0 (0.0) | 1 (10.0) | 0.926 |
|  | COPD/Asthma | 2 (9.1) | 1 (8.3) | 1 (10.0) | 1 |
| **c) Clinical Information** | | | | |  |
| Baseline diastolic blood pressure, mm Hg - median (IQR) | | 65.5 (61.5-74) | 67.5 (62.5-72) | 65.5 (61.75-73.25) | 0.766 |
| Baseline systolic blood pressure, mm Hg - median (IQR) | | 127 (121-139.75) | 138.5 (117.5-144) | 124 (121.75-129) | 0.137 |
|  | Normotensive (<130) - no. (%) | 12 (54.5) | 5 (41.7) | 7 (70.0) | 0.369 |
|  | Hypertensive (≥130) - no. (%) | 10 (45.5) | 7 (58.3) | 3 (30.0) |  |
| Baseline O_2_ saturation on room air, % - median (IQR) | | 0.88 (0.84-0.9) | 0.86 (0.8-0.88) | 0.88 (0.85-0.9) | 0.185 |
|  | Hypoxic (<90) - no. (%) | 16 (72.7) | 10 (83.3) | 6 (60.0) | 0.458 |
|  | Non-hypoxic (≥90) - no. (%) | 6 (27.3) | 2 (16.7) | 4 (40.0) |  |
| Baseline respiratory rate, respirations/min - median (IQR) | | 20 (18-25.5) | 22 (19.5-28.5) | 19 (16.5-21.5) | 0.053 |
|  | Eupnic (≤20) - no. (%) | 13 (59.1) | 6 (50.0) | 7 (70.0) | 0.607 |
|  | Tachypnic (>20) - no. (%) | 9 (40.9) | 6 (50.0) | 3 (30.0) |  |
| Baseline heart rate, beats/min - median (IQR) | | 82.5 (67-91.75) | 82.5 (73-91.25) | 77.5 (66.25-92.5) | 0.817 |
|  | Eucardic (≤100) - no. (%) | 22 (100.0) | 12 (100.0) | 10 (100.0) | n/a |
|  | Tachycardic (>100) - no. (%) | 0 (0.0) | 0 (0.0) | 0 (0.0) |  |
| Baseline temperature, °C - median (IQR) | | 37 (36.8-37.38) | 36.95 (36.77-37.12) | 37.15 (36.82-37.55) | 0.466 |
|  | Febrile (>37.0) - no. (%) | 9 (40.9) | 4 (33.3) | 5 (50.0) | 0.722 |
|  | Non-febrile (≤37.0) - no. (%) | 13 (59.1) | 8 (66.7) | 5 (50.0) |  |
| Baseline RBC Count, M/µL - median (IQR) | | 4.61 (4.36-4.94) | 4.61 (4.26-4.94) | 4.62 (4.44-4.94) | 0.598 |
|  | Low (<4.3) - no. (%) | 4 (18.2) | 3 (25.0) | 1 (10.0) | 0.541 |
|  | Normal (4.3-5.5) - no. (%) | 15 (68.2) | 8 (66.7) | 7 (70.0) |  |
|  | Elevated ( >5.5) - no. (%) | 3 (13.6) | 1 (8.3) | 2 (20.0) |  |
| Baseline WBC Count, K/µL - median (IQR) | | 5.75 (5.23-8.07) | 6.79 (5.34-7.98) | 5.62 (4.85-8.78) | 0.429 |
|  | Low (<4.5) - no. (%) | 2 (9.1) | 0 (0.0) | 2 (20.0) | 0.124 |
|  | Normal (4.5-11.0) - no. (%) | 19 (86.4) | 12 (100.0) | 7 (70.0) |  |
|  | Elevated ( >11.0) - no. (%) | 1 (4.5) | 0 (0.0) | 1 (10.0) |  |
| Baseline Neutrophils, K/µL - median (IQR) | | 4.43 (3.9-6.8) | 4.93 (4.3-6.38) | 4.03 (3.15-6.75) | 0.323 |
| Baseline Lymphocytes, K/µL - median (IQR) | | 1.04 (0.76-1.33) | 1.14 (0.69-1.6) | 0.92 (0.85-1.21) | 0.921 |
| Baseline Monocytes, K/µL - median (IQR) | | 0.36 (0.29-0.57) | 0.32 (0.25-0.5) | 0.38 (0.34-0.57) | 0.235 |
| Baseline Eosinophils, K/µL - median (IQR) | | 0.04 (0.04-0.06) | 0.04 (0.04-0.04) | 0.04 (0.04-0.06) | 0.129 |
| Baseline Basophils, K/µL - median (IQR) | | 0.04 (0.04-0.04) | 0.04 (0.04-0.04) | 0.04 (0.04-0.04) | 0.097 |
| Baseline Hemoglobin, g/dL - median (IQR) | | 13.4 (12.95-14) | 13.3 (12.7-14.15) | 13.45 (13.17-13.67) | 0.575 |
|  | Low (<13.2) - no. (%) | 8 (36.4) | 5 (41.7) | 3 (30.0) | 0.263 |
|  | Normal (13.2-16.4) - no. (%) | 12 (54.5) | 5 (41.7) | 7 (70.0) |  |
|  | Elevated ( >16.4) - no. (%) | 2 (9.1) | 2 (16.7) | 0 (0.0) |  |
| Baseline Platelet Count, K/µL - median (IQR) | | 224.5 (187.75-253) | 224 (171.75-251) | 224.5 (208.25-268.75) | 0.621 |
|  | Normal (150-450) - no. (%) | 21 (95.5) | 11 (91.7) | 10 (100.0) | 1 |
|  | Elevated ( >450) - no. (%) | 1 (4.5) | 1 (8.3) | 0 (0.0) |  |
| Baseline D-dimer, µg/mL - median (IQR) | | 0.95 (0.58-1.92) | 1.12 (0.69-1.65) | 0.79 (0.45-1.82) | 0.276 |
|  | Normal (<0.50) - no. (%) | 5 (22.7) | 1 (8.3) | 4 (40.0) | 0.21 |
|  | Elevated (≥0.50) - no. (%) | 17 (77.3) | 11 (91.7) | 6 (60.0) |  |
| Baseline International Normalized Ratio, sec - median (IQR) | | 1.1 (1-1.1) | 1.1 (1-1.15) | 1.1 (1-1.1) | 0.487 |
|  | Normal (0.9-1.1) - no. (%) | 17 (77.3) | 9 (75.0) | 8 (80.0) | 1 |
|  | Elevated (>1.1) - no. (%) | 5 (22.7) | 3 (25.0) | 2 (20.0) |  |
| Baseline ESR, mm/hr - median (IQR) | | 51.5 (40-71) | 43 (35.75-61.5) | 64.5 (43.75-71) | 0.198 |
| Baseline CRP, mg/L - median (IQR) | | 80.59 (68.91-142.69) | 80.59 (67.83-147.37) | 90.22 (72.53-132.12) | 0.895 |
| Baseline Troponin, ng/mL - median (IQR) | | 0.01 (0.01-0.02) | 0.01 (0.01-0.01) | 0.01 (0.01-0.03) | 0.193 |
| Time from symptom onset to infusion, days - median (IQR) | | 10.5 (8.25-12.75) | 10.5 (8.75-13) | 10.5 (8.25-11) | 0.571 |
|  | Earlier (≤10) - no. (%) | 11 (50.0) | 6 (50.0) | 5 (50.0) | 1 |
|  | Later (>10) - no. (%) | 11 (50.0) | 6 (50.0) | 5 (50.0) |  |
| Time from infusion to discharge, days - median (IQR) | | 6 (4-8.75) | 6 (3.75-9) | 5.5 (4-7.5) | 0.618 |
|  | Shorter (≤7) - no. (%) | 15 (68.2) | 8 (66.7) | 7 (70.0) | 1 |
|  | Longer (>7) - no. (%) | 7 (31.8) | 4 (33.3) | 3 (30.0) |  |
| Total hospital stay, days - median (IQR) | | 9 (6-11.75) | 9.5 (7.5-12.25) | 7 (6-10) | 0.371 |
|  | Shorter (≤10) - no. (%) | 15 (68.2) | 7 (58.3) | 8 (80.0) | 0.531 |
|  | Longer (>10) - no. (%) | 7 (31.8) | 5 (41.7) | 2 (20.0) |  |
| O_2_ requirement at admission, L/min - median (IQR) | | 2 (2-4) | 2 (2-8) | 2.5 (2-3.25) | 0.601 |
|  | None (<1) - no. (%) | 5 (22.7) | 3 (25.0) | 2 (20.0) | 0.598 |
|  | Low (1-49) - no. (%) | 16 (72.7) | 8 (66.7) | 8 (80.0) |  |
|  | High (≥50) - no. (%) | 1 (4.5) | 1 (8.3) | 0 (0.0) |  |
| Peak O_2_ requirement during hospital stay, L/min - median (IQR) | | 6.5 (3.25-11.25) | 8.5 (4.5-26.25) | 5 (3.25-6.75) | 0.119 |
|  | Low (1-49) - no. (%) | 18 (81.8) | 9 (75.0) | 9 (90.0) | 0.724 |
|  | High (≥50) - no. (%) | 4 (18.2) | 3 (25.0) | 1 (10.0) |  |
| O_2_ requirement at discharge, L/min - median (IQR) | | 3 (3-3) | 3 (3-3) | 3 (2-3) | 0.414 |
|  | None (<1) - no. (%) | 17 (77.3) | 10 (83.3) | 7 (70.0) | 0.816 |
|  | Low (1-49) - no. (%) | 5 (22.7) | 2 (16.7) | 3 (30.0) |  |
| ICU Stay - no. (%) | | 5 (22.7) | 4 (33.3) | 1 (10.0) | 0.43 |
| **d) Concomitant Medication** | | | | |  |
| Concurrent COVID-19 Treatments - no. (%) | |  |  |  |  |
|  | Convalescent Plasma | 19 (86.4) | 10 (83.3) | 9 (90.0) | 1 |
|  | Remdesivir | 19 (86.4) | 11 (91.7) | 8 (80.0) | 0.865 |
|  | Dexamethasone | 15 (68.2) | 9 (75.0) | 6 (60.0) | 0.77 |
|  | Anti-microbials | 16 (72.7) | 9 (75.0) | 7 (70.0) | 1 |

Median and Inter-quartile range (IQR) was determined for all continuous variables. P-values were obtained using Kruskal Wallis test continuous variables. Chi-square test was used to obtain p-values for categorical variables.

**Table S2. Immune Cell Marker Panels.**

| ***Cytek Flow Cytometry Panel*** | |
| --- | --- |
| **Marker** | **Description** |
| CD45RA | BUV395 Mouse Anti-Human CD45RA |
| Viability dye | LIVE/DEAD™ Fixable Blue Dead Cell Stain Kit, for UV excitation |
| CD16 | BUV496 Mouse Anti-Human CD16 |
| CCR5 | BUV563 Mouse Anti-Human CD195 (CCR5) |
| CD11c | BUV661 Mouse Anti-Human CD11c |
| CD56 | BUV737 Mouse Anti-Human CD56 |
| CD8 | BD Horizon™ BUV805 Mouse Anti-Human CD8 |
| CCR7 | Brilliant Violet 421™ anti-human CD197 (CCR7) Antibody |
| CD123 | CD123 Monoclonal Antibody (6H6), Super Bright 436, eBioscience™ |
| CD161 | CD161 Monoclonal Antibody (HP-3G10), eFluor 450, eBioscience™ |
| IgD | BV480 Mouse Anti-Human IgD |
| CD3 | Brilliant Violet 510™ anti-human CD3 Antibody |
| CD20 | CD20 Monoclonal Antibody (HI47), Pacific Orange |
| IgM | Brilliant Violet 570™ anti-human IgM Antibody |
| IgG | BD Horizon™ BV605 Mouse Anti-Human IgG |
| CD28 | Brilliant Violet 650™ anti-human CD28 Antibody (clone CD28.2) |
| CCR6 | Brilliant Violet 711™ anti-human CD196 (CCR6) Antibody |
| CXCR5 | BV750 Rat Anti-Human CXCR5 (CD185) |
| PD-1 | Brilliant Violet 785™ anti-human CD279 (PD-1) Antibody |
| CD141 | BD Horizon™ BB515 Mouse Anti-Human CD141 |
| CD57 | FITC anti-human CD57 Antibody |
| CD14 | Spark Blue™ 550 anti-human CD14 Antibody |
| CD45 | CD45 Monoclonal Antibody (H130), PerCP |
| CD11b | PerCP/Cyanine5.5 anti-human CD11b Antibody |
| TCR gd | TCR gamma/delta Monoclonal Antibody (B1.1), PerCP-eFluor 710, eBioscience™ |
| CD25 | CD25 Monoclonal Antibody (BC96), PE, eBioscience™ |
| CD4 | cFluor 568 Anti-human CD4 |
| CD24 | CD24 Monoclonal Antibody (eBioSN3 (SN3 A5-2H10)), PE-eFluor 610, eBioscience™ |
| CD95 | CD95 (APO-1/Fas) Monoclonal Antibody (DX2), PE-Cyanine5, eBioscience™ |
| CXCR3 | CD183 (CXCR3) Monoclonal Antibody (CEW33D), PE-Cyanine7, eBioscience™ |
| CD27 | CD27 Monoclonal Antibody (O323), APC, eBioscience™ |
| CD1c | Alexa Fluor® 647 anti-human CD1c Antibody |
| CD19 | Spark NIR™ 685 anti-human CD19 Antibody |
| CD127 | APC-R700 Mouse Anti-Human CD127 |
| HLA-DR | HLA-DR Monoclonal Antibody (L243), APC-eFluor 780, eBioscience™ |
| CD38 | CD38 APC-Fire810 |
| ***Immune Monitoring Cytometry Panel*** | |
| **Marker** | **Description** |
| Viability dye | LIVE/DEAD™ Fixable Blue Dead Cell Stain Kit, for UV excitation |
| CD45 | CD45 Monoclonal Antibody (2D1), Super Bright 645, eBioscience™ |
| CD3 | BUV395 Mouse Anti-Human CD3 Clone SK7 |
| CD8 | CD8a Monoclonal Antibody (OKT8 (OKT-8)), Super Bright 436, eBioscience™ |
| CD4 | CD4 Monoclonal Antibody (RPA-T4), PerCP-Cyanine5.5, eBioscience™ |
| FOXP3 | FOXP3 Monoclonal Antibody (PCH101), eFluor 450, eBioscience™ |
| CD11b | BUV661 Rat Anti-CD11b Clone M1/70 |
| CD56 | Brilliant Violet 750™ anti-human CD56 (NCAM) Antibody |
| CD45RO | BB515 Mouse Anti-Human CD45RO Clone UCHL1 |
| CD25 | CD25 Monoclonal Antibody (BC96), Super Bright 600, eBioscience™ |
| PD1 | BUV737 Mouse Anti-Human CD279 (PD-1) Clone EH12.1 |
| Tim3 | CD366 (TIM3) Monoclonal Antibody (F38-2E2), Super Bright 702, eBioscience™ |
| TOX | TOX Antibody, anti-human/mouse, APC, REAfinity™ |
| TCF1 | PE anti-TCF1 (TCF7) Antibody |
| CD44 | APC/Cyanine7 anti-mouse/human CD44 Antibody |
| CD62L | BV421 Mouse Anti-Human CD62L Clone DREG-56 |
| CTLA4 | PE/Dazzle™ 594 anti-human CD152 (CTLA-4) Antibody |
| Lag-3 | CD223 (LAG-3) Monoclonal Antibody (3DS223H), PE-Cyanine5, eBioscience™ |
| Klrg1 | Brilliant Violet 510™ anti-mouse/human KLRG1 (MAFA) Antibody |
| T-bet | BV786 Mouse Anti-T-bet Clone O4-46 |
| Ki-67 | Ki-67 Monoclonal Antibody (SolA15), PerCP-eFluor 710, eBioscience™ |
| GzmB | Granzyme B Monoclonal Antibody (N4TL33), Alexa Fluor 532, eBioscience™ |
| VISTA | VISTA Monoclonal Antibody (B7H5DS8), PE-Cyanine7, eBioscience™ |
| ICOS | Alexa Fluor® 488 anti-human/mouse/rat CD278 (ICOS) Antibody |
| CD69 | BUV805 Mouse Anti-Human CD69 Clone FN50 |

**Table S5. First principal component (PC1) loadings of each activation marker were used as coefficients for defining the activation score.**

| **Marker** | **PC1 loading**  **for HD & COVID D1** | **Average Log-Fold Change**  **(HD vs. COVID day 1)** | **Wilcoxon p-value**  **(HD vs. COVID day 1)** |
| --- | --- | --- | --- |
| ***CD8+ T cells*** | | | |
| **T-bet** | 0.71 | 0.52 | <0.001 |
| **Ki-67** | 0.39 | 0.46 | <0.001 |
| **CD69** | 0.28 | 0.39 | <0.001 |
| **TOX** | 0.40 | 0.31 | <0.001 |
| **GZMB** | 0.31 | 0.20 | <0.001 |
| ***CD4+ T cells (total)*** | | | |
| **T-bet** | 0.14 | 0.08 | <0.001 |
| **Ki67** | 0.69 | 0.38 | <0.001 |
| **CD69** | 0.34 | 0.41 | <0.001 |
| **TOX** | 0.33 | 0.19 | <0.001 |
| **PD1** | 0.53 | 0.11 | <0.001 |
| ***Treg cells*** | | | |
| **Ki-67** | 0.76 | 0.33 | <0.001 |
| **TOX** | 0.14 | 0.38 | <0.001 |
| **CD25** | 0.07 | 0.09 | <0.001 |
| **iCOS** | 0.43 | 0.17 | <0.001 |
| **CTLA4** | 0.47 | 0.30 | <0.001 |
| ***NK cells*** | | | |
| **TOX** | 0.16 | 0.42 | <0.001 |
| **GZMB** | 0.07 | 0.28 | <0.001 |
| **KLRG1** | 0.08 | 0.06 | <0.001 |
| **Ki-67** | 0.89 | 0.84 | <0.001 |
| **LAG3** | 0.03 | 0.08 | <0.001 |

**Table S6. Centrality ranks of filtered and weighted correlations.**

| **Cytokine markers** | **HD** | **D1** | **Placebo** | **CD24Fc** | **var** | **Mean** |
| --- | --- | --- | --- | --- | --- | --- |
| IL-5 | 1 | 1 | 1 | 9 | 16 | 3 |
| MIP-1d | 17 | 2 | 12 | 1 | 60·67 | 8 |
| IL-1b | 11 | 3 | 2 | 2 | 19 | 4·5 |
| IL-8 | 2 | 4 | 8 | 17 | 44·25 | 7·75 |
| G-CSF | 20 | 5 | 9 | 4 | 53·67 | 9·5 |
| IL-16 | 15 | 6 | 4 | 6 | 24·25 | 7·75 |
| MIG | 16 | 7 | 17 | 5 | 37·58 | 11·25 |
| IL-4 | 10 | 8 | 6 | 3 | 8·92 | 6·75 |
| MCSF | 24 | 9 | 13 | 16 | 40·33 | 15·5 |
| IL-12p40 | 14 | 10 | 5 | 7 | 15·33 | 9 |
| IL-15 | 13 | 11 | 7 | 8 | 7·58 | 9·75 |
| IL-1a | 7 | 12 | 11 | 12 | 5·67 | 10·5 |
| TNF RI | 25 | 13 | 14 | 14 | 32·33 | 16·5 |
| I-309 | 8 | 14 | 3 | 11 | 22 | 9 |
| MIP-1a | 12 | 15 | 21 | 13 | 16·25 | 15·25 |
| BLC (CXCL13) | 27 | 16 | 25 | 15 | 37·58 | 20·75 |
| TNF RII | 22 | 17 | 18 | 21 | 5·67 | 19·5 |
| IL-6sR | 30 | 18 | 28 | 22 | 30·33 | 24·5 |
| IL-7 | 5 | 19 | 19 | 24 | 66·92 | 16·75 |
| MIP-1b | 19 | 20 | 27 | 30 | 28·67 | 24 |
| IL-6 | 6 | 21 | 20 | 28 | 84·92 | 18·75 |
| PDGF-BB | 9 | 21 | 22 | 18 | 35 | 17·5 |
| RANTES | 18 | 23 | 23 | 19 | 6·92 | 20·75 |
| GM-CSF | 4 | 24 | 16 | 25 | 94·25 | 17·25 |
| TIMP-1 | 23 | 25 | 24 | 20 | 4·67 | 23 |
| IL-10 | 3 | 26 | 15 | 26 | 120·33 | 17·5 |
| Eotaxin-2 (CCL24) | 30 | 27 | 30 | 30 | 2·25 | 29·25 |
| ICAM-1 | 30 | 28 | 26 | 23 | 8·92 | 26·75 |
| Eotaxin (CCL11) | 26 | 29 | 10 | 10 | 103·58 | 18·75 |
| TIMP-2 | 21 | 30 | 29 | 27 | 16·25 | 26·75 |
| HD, Healthy donor; D1, baseline COVID-19 patients; Var, variance.  Centrality scores ranked from highest (1, red) to lowest (30, blue). Variance and means calculated based on rank. | | | | | | |
